# Physicochemical Characterization and Antioxidant Activity Evaluation of Idebenone/Hydroxypropyl-*β*-Cyclodextrin Inclusion Complex †

**DOI:** 10.3390/biom9100531

**Published:** 2019-09-25

**Authors:** Valentina Venuti, Vincenza Crupi, Barbara Fazio, Domenico Majolino, Giuseppe Acri, Barbara Testagrossa, Rosanna Stancanelli, Federica De Gaetano, Agnese Gagliardi, Donatella Paolino, Giuseppe Floresta, Venerando Pistarà, Antonio Rescifina, Cinzia A. Ventura

**Affiliations:** 1 Dipartimento di Scienze Matematiche e Informatiche, Scienze Fisiche e Scienze della Terra, Università degli Studi di Messina, V.le F. Stagno D’Alcontres, 31-98166 Messina, Italy; vvenuti@unime.it; 2 Dipartimento di Scienze Chimiche, Biologiche, Farmaceutiche e Ambientali, Università degli Studi di Messina, V.le F. Stagno D’Alcontrés, 31-98166 Messina, Italy; vcrupi@unime.it (V.C.); rstancanelli@unime.it (R.S.); fedegaetano@unime.it (F.D.G.); 3 CNR-IPCF Istituto per i Processi Chimico Fisici, V.le F. Stagno d’Alcontres, 37-98158 Faro Superiore, Messina, Italy; fazio@me.cnr.it; 4 Dipartimento di Scienze Biomediche, Odontoiatriche, e delle Immagini Morfologiche e Funzionali, Università degli Studi di Messina, c/o A.O.U. Policlinico “G. Martino” Via Consolare Valeria, 1-98125 Messina, Italy; gacri@unime.it (G.A.); btestagrossa@unime.it (B.T.); 5 Dipartimento di Medicina Clinica e Sperimentale, Università degli Studi di Catanzaro “Magna Græcia”, Campus Universitario “S. Venuta”, Viale S. Venuta-88100 Germaneto, Catanzaro, Italy; gagliardi@unicz.it (A.G.); paolino@unicz.it (D.P.); 6 Dipartimento di Scienze del Farmaco, Università degli Studi di Catania, V.le A. Doria, 6-95125 Catania, Italy; giuseppe.floresta@unict.it (G.F.); vpistara@unict.it (V.P.); arescifina@unict.it (A.R.)

**Keywords:** idebenone, (2-hydroxypropyl)-*β*-cyclodextrin, inclusion complex, physicochemical characterization, antioxidant activity, U373 cells, excised bovine nasal mucosa

## Abstract

Idebenone (IDE) is an antioxidant drug active at the level of the central nervous system (CNS), whose poor water solubility limits its clinical application. An IDE/2-hydroxypropyl-*β*-cyclodextrin (IDE/HP-*β*-CD) inclusion complex was investigated by combining experimental methods and theoretical approaches. Furthermore, biological in vitro/ex vivo assays were performed. Phase solubility studies showed an A_L_ type diagram, suggesting the presence of a 1:1 complex with high solubility. Scanning electron microscopy (SEM) allowed us to detect the morphological changes upon complexation. The intermolecular interactions stabilizing the inclusion complex were experimentally characterized by exploring the complementarity of Fourier-transform infrared spectroscopy in attenuated total reflectance geometry (FTIR-ATR) with mid-infrared light, Fourier-transform near-infrared (FT-NIR) spectroscopy, and Raman spectroscopy. From the temperature evolution of the O–H stretching band of the complex, the average enthalpy Δ*H*_HB_ of the hydrogen bond scheme upon inclusion was obtained. Two-dimensional (2D) rotating frame Overhauser effect spectroscopy (ROESY) analysis and computational studies involving molecular modeling and molecular dynamics (MD) simulation demonstrated the inclusion of the quinone ring of IDE inside the CD ring. In vitro/ex vivo studies evidenced that complexation produces a protective effect of IDE against the H_2_O_2_-induced damage on human glioblastoma astrocytoma (U373) cells and increases IDE permeation through the excised bovine nasal mucosa.

## 1. Introduction

**Figure 1:**
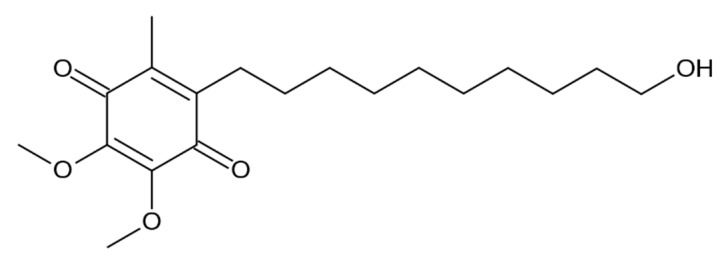
Chemical structure of 2,3-dimethoxy-5-methyl-6-(10-idroxydecyl)-1,4-benzoquinone (IDE).

Idebenone (2,3-dimethoxy-5-methyl-6-(10-idroxydecyl)-1,4-benzoquinone; IDE, Figure 1) is an analog of coenzyme Q10 (CoQ10), a vital cell membrane antioxidant and an essential part of the cellular machinery used to produce adenosine triphosphate (ATP) [1]. It is a potent antioxidant able to retard lipid peroxidation, thereby protecting mitochondria against oxidative stress. Furthermore, IDE improves mitochondrial respiratory chain function, facilitating ATP production [2,3].

IDE is a drug active at the level of the central nervous system (CNS), and shows beneficial effects in a wide range of neurological disorders including trauma, cerebral ischemia, and hypertension-induced vascular lesions [4,5]. IDE is also considered an attractive molecule for skincare and anti-aging treatments [6].

Takeda Pharmaceuticals initially developed IDE for the treatment of cognitive disturbances and Alzheimer’s disease [7]. The drug was approved in Japan in 1986; nevertheless, clinical trials later demonstrated that IDE was ineffective toward patients with Alzheimer’s disease [8]. As a consequence, IDE was withdrawn from the Japanese market in 1998. IDE was introduced in 1993 into the Italian market for the treatment of cognitive disturbances resulting from cerebral pathologies of both vascular and degenerative origin, at a dose of 90 mg/day (MNESIS) [9]. IDE as Raxone was approved in Europe in 2015 to treat visual impairment in adolescents (12 years and older) and adults with Leber’s hereditary optic neuropathy (LHON) [10,11,12], an inherited disease produced by mutations in the mitochondrial DNA, leading to acute or subacute bilateral visual loss.

For its ability to enhance the flux of electrons along the electron transport chain (ETC), thereby facilitating the generation of ATP, IDE was postulated as a potential therapeutic agent for Friederich’s ataxia (FA) [13]. This disorder is caused by mutations in the gene that encodes for frataxin. Lack of this protein impairs the energy production in the mitochondria and damages nervous and cardiac tissues. However, contradictory results were obtained regarding the efficacy of IDE in treating FA patients [13,14,15,16]. Then, after a first “conditioned” authorization, in 2008, of IDE (CATENA) in Canada, a phase III study (MICONOS) by Santhera Pharmaceuticals missed its primary endpoint and the drug was withdrawn from the market in 2013 for lack of efficacy [17]. A phase III clinical trial (DELOS study) demonstrated that Raxone can slow a loss of respiratory function and reduce bronchopulmonary complications in patients with Duchenne muscular dystrophy (DMD) who did not undergo a simultaneous treatment with glucocorticoids [18,19]. A long term (six years) study (SYROS study) confirmed these results. At present, Santhera Pharmaceuticals is evaluating Raxone efficacy in patients with DMD receiving glucocorticoid steroids (phase III—SIDEROS study). Results are expected in 2020.

IDE is a poorly water-soluble drug, and tablets for oral administration are marketed. After absorption, IDE is rapidly transformed by first-pass metabolism into inactive metabolites, and only 1% of the oral administered dose reaches the systemic circulation [20]. The erratic oral bioavailability and the high affinity of IDE for plasma proteins could limit its brain accumulation, thus negatively affecting its therapeutic potentialities. Dosage forms different from tablets may be needed.

Intranasal administration of drugs represents a suitable way to obtain nose-to-brain targeting [21], by granting direct access to the brain via the olfactory and trigeminal nerve pathways. In this way, the blood–brain barrier (BBB) and the pre-systemic gastrointestinal and hepatic elimination are circumvented [22]. However, many physiological factors can reduce the brain bioavailability of drugs [23]. As a consequence, different drug delivery approaches were attempted to increase the transport through nasal mucosa [22]. Among them, drug–cyclodextrin complexes were proven to increase aqueous solubility, dissolution rate, and bioavailability of lipophilic drugs, as well as acting as penetration enhancers through biological membranes [24,25,26,27,28]. Moreover, cyclodextrins (CDs) can reduce or prevent degradation of drugs by enzymes, with these being largely present on the nasal mucosa surface [29].

Starting from 20 years ago, we performed in solution studies and solid-state characterization of inclusion complexes of IDE with native and modified *β*-CD [30,31]. We fully characterized an IDE/sulfobutyl ether-*β*-cyclodextrin (IDE/SBE-*β*-CD) inclusion complex intended for oral administration, evidencing an increase of IDE water solubility because of complexation (from about 0.008 mg/mL for the free drug to about 0.3 mg/mL for the complex), and a subsequent enhancement of IDE dissolution rate (100% complex dissolved within 180 min, whereas about 5% free IDE dissolved in the same time interval) [30]. Now, our research addresses the development of an inhalable powder, with penetration enhancer properties, for nose-to-brain targeting. After inhalation, the formulation should promptly dissolve to guarantee a fast penetration of solubilized IDE. With this aim in mind, we chose to investigate the inclusion complex of IDE with 2-hydroxypropyl-*β*-cyclodextrin (HP-*β*-CD), since it was already shown to exhibit a higher water solubility (about 600 times) [31] with respect to the IDE/SBE-*β*-CD system. The ability of the IDE/HP-*β*-CD complex to prevent carrageenan-induced hyperalgesia and edema was assayed and results were previously published [32]; however, we did not perform any physicochemical investigation, and no results were found in the literature. Furthermore, even if some authors reported on the increase in antioxidant activity of different drugs via complexation with CDs [33], no studies concerning the in vitro antioxidant activity of the IDE/HP-*β*-CD inclusion complex are reported in literature; thus, we decided to go in depth in this respect. Finally, on the basis of the demonstrated ability of HP-*β*-CD to complex cholesterol and to interact with the hydrophilic components of membranes [34], an enhancement of the drug permeation across nasal mucosa could be forecast. This, together with the increase in IDE antioxidant activity, also expected, could efficiently improve the therapeutic performance of the investigated drug.

A water-soluble formulation of IDE based on HP-*β*-CD complexation was developed, and a multi-technique investigation of its physicochemical properties was performed. In the liquid state, phase solubility studies according to the method reported by Higuchi and Connors [35] were performed. Furthermore, one- and two-dimensional nuclear magnetic resonance (NMR) spectroscopy measurements were carried out to investigate the host–guest interactions and the spatial disposition of IDE into the CD cavity.

The morphology of the complex in the solid state was characterized by scanning electron microscopy (SEM), whereas the combined use of Fourier-transform infrared spectroscopy in attenuated total reflectance geometry (FTIR-ATR) with mid-infrared light, Fourier-transform near-infrared (FT-NIR) spectroscopy, and Raman spectroscopy allowed for deep characterization of the changes in the vibrational features of the functional groups involved in the host–guest interactions that occur in the complex. In order to achieve an energetic and structural rationalization of the recognition process, molecular modeling and molecular dynamics (MD) simulation studies on the IDE/HP-*β*-CD complex were conducted. Furthermore, to investigate the influence of the CD on the antioxidant activity of IDE, preliminary in vitro studies on human glioblastoma astrocytoma (U373) cells were performed in comparison with the free drug. Finally, ex vivo studies on excised bovine nasal mucosa were carried out to probe the ability of complexed IDE to cross biomembranes.

## 2. Materials and Methods

### 2.1. Materials

Idebenone (IDE, C_19_H_30_O_5_, molecular weight (MW) 338.44) and (2-hydroxypropyl)-*β*-cyclodextrin (HP-*β*-CD, 0.6 molar substitution, average MW 1380) were purchased from Sigma-Aldrich (St. Louis, MO, USA). Dulbecco’s modified Eagle’s medium (DMEM), heat-inactivated fetal bovine serum (FBS), penicillin/streptomycin solution, glutamine, and trypsin were obtained from GIBCO (Invitrogen Corporation, Giuliano Milanese, Milano, Italy). Plastic culture flasks and disposable cell filters were obtained from Costar (Cambridge, MA, USA). Double-distilled pyrogenic-free water hydrogen peroxide and sterile saline solution were Sifra S.p.A. (Verona, Italy) and Fresenius Kabi Potenza S.r.l. (Verona, Italy) products, respectively. Human glioblastoma astrocytoma (U373) cells were purchased from ATCC (American Type Culture Collection) retailer LGC Standards S.r.l. (Sesto San Giovanni, Milano, Italy). The Pierce lactic dehydrogenase (LDH) cytotoxicity assay kit was obtained from Thermo Fisher-Scientific (USA). All other products and reagents were of analytical grade.

### 2.2. Preparation of the Physical Mixture and the Inclusion Complex

An IDE + HP-*β*-CD physical mixture in a 1:2 molar ratio was obtained by carefully mixing an accurately weighed amount of IDE and HP-*β*-CD, until the mixture was homogeneous.

The IDE/HP-*β*-CD inclusion complex was prepared using the freeze-drying method in a 1:2 molar ratio. Briefly, HP-*β*-CD (8.28 g, 6 × 10^−3^ M) was solubilized, at room temperature, in water (80 mL), in a capped tube; then, a methanol solution (20 mL) containing IDE (1 g, 3 × 10^−3^ M) was added. The tube was sealed to avoid changes due to evaporation, and the solution was magnetically stirred at room temperature for 12 h. The solution was poured into freeze-drying flasks and placed into the vacuum chamber, frozen at −40 °C, and then freeze-dried for 72 h (VirTis Gardiner, USA BenchTop K Series Freeze Dryers).

### 2.3. Scanning Microscope Electronic Measurements

The microstructure (morphology) of the composite particles was observed employing scanning electron microscopy (SEM) (JMC-6000 Jeol, Japan). The SEM measurements were conducted at 15 kV using a magnification of 130×. Samples were mounted on adhesive black carbon tabs, pre-mounted on the specimen holder.

### 2.4. FTIR-ATR Spectroscopy Measurements

FTIR-ATR spectra were collected on solid samples, in the mid-infrared (MIR) wavenumber region (4000–400 cm^−1^) and in the 250–340 K temperature range. A Bomem DA8 Fourier-transform spectrometer was used, operating with a Globar source, combined with a KBr beamsplitter and a thermoelectrically cooled deuterated triglycine sulfate (DTGS) detector. When FTIR measurements are performed in ATR geometry, the powder is placed in contact with the surface of the ATR crystal and pressed by a piston in diamond [36]. The evanescent wave is attenuated in that region of the IR spectrum where the sample absorbs energy. The main advantage of the FTIR-ATR technique is represented by the fact that the evanescent wave exponentially decays in the sample with the distance from the surface of the crystal, within a distance on the order of microns. Then, it is independent of the thickness of the adsorbed sample.

Experiments were performed in a dry atmosphere, with a resolution of 4 cm^−1^, automatically adding 100 repetitive scans to guarantee a good signal-to-noise ratio and high reproducibility. Spectra were normalized to take into account the effective number of absorbers. No smoothing was done, and Spectracalc software package GRAMS (Galactic Industries, Salem, NH, USA) was used for baseline adjustment and normalization. Band deconvolution of the 3800–3000 cm^−1^ range, to which the O–H stretching vibrational mode typically belongs, was performed through second-derivative computations, which were used to evaluate wavenumbers of the maxima of the different sub-bands.

Based on the obtained results, the experimental data were fitted by a multiple curve-fitting routine provided in the PeakFit 4.0 software package (Systat Software, Inc., USA). Voigt fitting functions were chosen, and all parameters (center frequency, amplitude, linewidth) were left free to vary upon iteration. For each fitting session, multiple iterations were performed until a converging solution was reached by minimization of the value of *R*^2^. The procedure used allows applying the minimum number of parameters during analysis. The best fit is characterized by an *R*^2^ ≈ 0.9999 for all samples.

### 2.5. FT-NIR Measurements

FT-NIR spectra were collected in the absorbance mode using the Antaris II FT-NIR spectrophotometer (Thermo Fischer Scientific, Waltham, MA, USA). Each spectrum was the average of 2048 scans, using air as the reference. The spectral range was 10,000–4000 cm^−1^, and the data were measured in 1.928-cm^−1^ intervals, which resulted in 3112 variables. In FT-NIR spectra collection, the near-infrared spectrophotometer was sensitive to the change in outer environment conditions such as temperature and humidity. Therefore, the temperature was kept at 25 °C, and the humidity was kept at a steady level in the laboratory.

### 2.6. Raman Spectroscopy Measurements

Raman experiments were acquired using a LabRam HR800 Micro-Raman Spectrometer (Horiba Jobin Yvon, Kyoto, Japan) equipped with an Olympus BX41-microscope (Olympus, Tokyo, Japan) working in a backscattering configuration. The HR800 employs a solid-state laser source (*λ* = 561 nm). The laser beam is focused employing a 50× long working distance microscope objective (Olympus LMPlanFl 50× (Olympus, Tokyo, Japan), numerical aperture (NA) 0.5, working distance (WD) = 10.6 mm) on a ~1-μm-diameter spot. The laser power on the sample is 5 mW. The Raman signals are collected via the same illumination objective, in backscattering configuration, dispersed by a 600 lines/mm grating and detected through a Peltier-cooled silicon CCD (Synapse by Horiba Jobin Yvon). Spectra were recorded in the 4000–150 cm^−1^ range with a resolution of 1.27 cm^−1^, and typically acquired with integration times of 30 s.

### 2.7. Phase-Solubility Measurements

The analyses were conducted employing a thermostated bath (Telesystem 15.40, Thermo Scientific, USA) with a temperature control unit, which allows having an accuracy of 0.01 °C (Telemodul 40C, Thermo Scientific, USA). The exact amount of IDE (10 × 10^−3^ M), which exceeds its intrinsic solubility (~2 × 10^−5^ M), was added to non-buffered aqueous solutions with increasing concentrations of HP-*β*-CD from 0 mM to 9 mM in 10-mL capped tubes, then sonicated in a Bandelin RK 514 water bath (Berlin, Germany) for 15 min. Suspensions were placed in the thermostat bath, at 25.0 ± 0.5 °C, under magnetic stirring for 48 h. Then, the suspensions were filtered through Sartorius Minisart-SRP 15 polytetrafluoroethylene (PTFE) 0.22-μm filters (Germany) and analyzed by High Performance Liquid Chromatography (HPLC) to evaluate the amount of dissolved IDE. Experiments were carried out in triplicate. Data obtained from the phase solubility diagram were used to calculate the association constant (*K*_c_) of the IDE/HP-*β*-CD inclusion complex according to the following equation: *K*_c_ = *Slope*/*S*_0_ × (1 − *Slope*) [35], where *S*_0_ is the intrinsic water solubility of IDE.

### 2.8. Titration Studies

Ultraviolet–visible light (UV–Vis) spectra were performed in the 200–400 nm spectral range by a FullTech Instruments (Roma, Italy) double-beam spectrophotometer mod PG T80 (resolution 0.001 × 10^−3^ absorbance units; signal-to-noise ratio, 1 × 10^−4^). Free IDE (4.43 × 10^−5^ M) guest (G), or in the presence of different HP-*β*-CD host (H) concentrations (4.43 × 10^−5^ M, 8.86 × 10^−5^ M, 1.33 × 10^−4^ M, 2.21 × 10^−4^ M, 4.43 × 10^−4^ M, 6.64 × 10^−4^ M, 2.21 × 10^−3^ M), was solubilized in a water/methanol solution (90/10, *v*/*v*) and stirred before the analysis for 12 h. The absorbance data of the titration experiment were analyzed using the HypSpec software [37,38,39]. Different binding equilibria between the two species were considered. The experimental data were either applied to a single equilibrium model 1:1 (H:G) or multiple binding equilibria models such as a 1:1 (H:G) + 1:2 (H:G) model and/or a 1:1 (H:G) + 2:1 (H:G) model for best data fit with HypSpec. The experimental data were tested against all the different types of binding models to establish a proper stoichiometry and association constants for all studied cases.

### 2.9. In Vitro Dissolution Studies

In vitro dissolution studies were conducted by following the United States Pharmacopoeia (USP) 32nd paddle method. Free or complexed IDE was suspended in 900 mL of water and kept under magnetic stirring (100 rpm) at 25 ± 0.5 °C. Aliquots of the suspension were taken at predetermined time intervals (from time zero to 180 min) and analyzed by HPLC to determine the amount of IDE in solution. After each sampling, the solutions were replenished with fresh preheated water, and data obtained from HPLC were corrected for the dilution. Sink conditions were maintained for all the experiments.

### 2.10. HPLC Analysis and Method Validation

The quantitative analysis of IDE was carried out by reverse-phase HPLC (HPLC Prominence LC-20AB pump, Shimadzu, North America). Twenty microliters of each sample was injected into the Discovery C18 column (250 × 4.6 mm inner diameter (i.d.), 5 μm, Supelco). The flow rate was fixed at 1 mL/min and the mobile phase consisted of water/acetonitrile (25/75, *v*/*v*). IDE was detected at 280 nm using a UV–Vis detector (Shimadzu UV–Vis detector SPD-20A).

The HPLC method was validated for linearity, specificity, sensitivity, and repeatability. A stock solution of IDE was prepared by dissolving 10 mg in 100 mL of a water/acetonitrile mixture (25/75, *v*/*v*) into a calibrated flask. Calibration standards were prepared at concentrations ranging from 0.5 μg/mL to 50 μg/mL, starting from stock solution. Six replicates of each concentration were prepared and analyzed in triplicate. To determine specificity, a calibration standard containing 1 μg/mL of IDE was added to different amounts of HP-*β*-CD (IDE/HP-*β*-CD molar ratio: 1:1, 1:2, 1:20) and analyzed in triplicate. Limit of detection (LOD) and limit of quantitation (LOQ) were determined by signal-to-noise ratio [40]. The precision of the method was determined intraday and interday by injecting three of the calibration standards (0.5, 10, 50 μg/mL) six times during the same day and the next five days (see Analytical Method Validation in Supplementary Materials, Figure S1).

### 2.11. Nuclear Magnetic Resonance Measurements

Samples of equivalent concentrations (14 mM) of IDE, HP-*β*-CD, and IDE/HP-*β*-CD inclusion complex were prepared in a D_2_O/CD_3_OD (1:1, *v*/*v*) solution and transferred to 5-mm NMR tubes for spectrum acquisition. All spectra were recorded at 300 K with a Varian Unity Inova 500 MHz (11.75 T) instrument. The residual semiheavy water HDO (4.70 ppm) was used as internal reference, to avoid the addition of external ones that could interact with HP-*β*-CD. Spectra using diffusion ordered spectroscopy (DOSY) were recorded using the DgcsteSL_cc (DOSY gradient compensated stimulated echo with spin-lock and convection compensation) high-resolution diffusion ordered spectroscopy HR-DOSY sequence. The pulsed gradient range amplitudes were 0.1067–0.5334 T/m, at a diffusion time of 0.06 s. The processing program (DOSY macro in the Varian instrument) was run with the data transformed using fn = 32 K and lb = 0.3. Rotating frame Overhauser effect spectroscopy (ROESY) spectra were recorded using the ROESYAD sequence (transverse cross-relaxation experiment in rotating frame with adiabatic mixing pulses) using a mixing time of 500 ms.

### 2.12. Structure Preparation

A three-dimensional (3D) structure for the HP-*β*-CD was not available; thus, the structure was built using *β*-CD obtained from the Protein Data Bank (PDB identifier (ID): 5E6Z). In the experimental section, we used an HP-*β*-CD with an average MW of 1380 and a molar substitution degree of 0.6, which corresponds to four 2-hydroxypropyl groups for a molecule of HP-*β*-CD. The actual substitution on the OH groups and configuration are unknown; thus, four glucopyranose units were substituted with the 2-hydroxypropyl group. Two different models were created: in the first one, four oxygens on the C_2_ position of the secondary rim (residues 1, 3, 5, and 7) were substituted with the 2-hydroxypropyl group, whereas, in the second one, the four oxygens on the C_6_ position of the primary rim (residues 1, 3, 5 and 7) were substituted. This assures a sufficient representation of the hindrance induced by the 2-hydroxypropyl groups [41]. The structures of CD and IDE were built using Marvin Sketch. A neutral pH for the protonation states of the molecules was considered. All the 3D structures were firstly subjected to a molecular mechanics energy minimization by Merck molecular force field; then, the MOPAC package (vMOPAC2016, Stewart Computational Chemistry, Colorado Springs, CO, USA) was used to fully optimize the geometry of structures at the semi-empirical level, using the parameterized model number 6 Hamiltonian.

### 2.13. Molecular Dynamics Simulations

The MD simulation was made in explicit water using YASARA (17.8.15) software [42,43]. A periodic simulation cell with boundaries extending 10 Å from the surface of the two molecules, IDE and HP-*β*-CD, separated by a distance of 5 Å, was employed. The box was filled with water, with a maximum sum of all bumps per water of 1.0 Å, and a density of 0.997 g/mL with explicit solvent. YASARA’s p*K*_a_ utility was used to assign p*K*_a_ values at pH 7.0. Waters were deleted to readjust the solvent density to 0.997 g/mL. The AMBER 14 force field was used with long-range electrostatic potentials calculated with the particle mesh Ewald (PME) method, with a cutoff of 8.0 Å [44]. The IDE and HP-*β*-CD force field parameters were generated with the AutoSMILES utility [45], which employs semiempirical AM1 geometry optimization and assignment of charges, followed by the assignment of the AM1BCC atom and bond types with refinement using the RESP charges, and finally the assignments of general AMBER force field atom types. A short MD simulation was run on the solvent only. The entire system was then energy minimized firstly using a steepest descent minimization to remove conformational stress, followed by a simulated annealing minimization until convergence (<0.01 kcal/mol∙Å). The MD simulation was then initiated, using the NVT ensemble at 298 K, and integration time steps for intramolecular and intermolecular forces every 1.25 fs and 2.5 fs, respectively. The MD simulation was stopped after 150 ns, and single snapshots were recorded every 250 ps. All simulations were performed without constraints.

### 2.14. Binding Free Energy Calculation

On the optimized MD structure obtained from the previous step, the binding free energy was calculated using the well-known and widely used molecular mechanics Poisson–Boltzmann surface area (MM/PBSA) approach implemented in YASARA, adopting the consolidate protocol of Nunthaboot [46,47].

### 2.15. Culture Cells

The U373 cells were incubated (Water-Jacketed CO2 Incubator, Thermo Scientific, Germany) in plastic culture dishes (100 mm × 20 mm) at 37.0 ± 0.1 °C (5% CO_2_) using DMEM medium with glutamine, enriched with penicillin (100 UI/mL), streptomycin (100 µg/mL), amphotericin B (250 µg/mL), and FBS (10% *v*/*v*), in order to promote their adhesion to the plate. Fresh medium was substituted every 48 h. When the cells reached about 80% confluence, trypsin (2 mL) was added for cell detaching. After that, the cells were collected into a centrifuge tube containing 4 mL of the culture medium. The dishes were further washed with 2 mL of phosphate buffer solution (PBS, pH 7.4) to remove the remaining cells; then, the PBS was transferred into the centrifuge tube. The tube was centrifuged at 1500 rpm at room temperature for 5 min using a Megafuge 1.0 (HeraeusSepatech, Osterode/Harz, Germany). The pellet was resuspended in an appropriate culture medium volume and seeded in culture dishes before in vitro investigation.

### 2.16. In Vitro Cytotoxicity Assays

To investigate the cytotoxicity of the IDE/HP-β-CD inclusion complex and free IDE, MTT (Methylthiazolyldiphenyl-tetrazolium bromide) testing was carried out [48]. The U373 cells were plated in 96-multiwell dishes (8 × 10^3^ cells/0.1 mL) and treated with different concentrations of free and complexed IDE (5, 10, 20, 30, and 40 µM), then incubated for 24, 48, or 72 h. After 3 h of incubation with tetrazolium salts, the percentage of cell viability was calculated by ELISA microplate reader (BIO-RAD, xMarkTMMicroplate Absorbance Spectrophotometer) at *λ*_abs_ 570 nm and *λ*_abs_ 670 nm according to the following Equation (1):

cell viability (%) = AbsT/AbsC × 100, (1)

where AbsT is the absorbance of treated cells, and AbsC is the absorbance of control (untreated) cells. The formazan concentration is correlated to the cell viability that was reported as the average of three different experiments ± standard deviation.

### 2.17. Evaluation of Antioxidant Activity

Antioxidant activity was assayed by measuring lactic dehydrogenase (LDH) release. As it is well known, this is a useful method for the detection of necrosis due to membrane rupture and subsequent cell disruption.

The U373 cells were placed in a 96-well culture dishes (8 × 10^3^ cells/0.1 mL) and treated with different concentrations of IDE alone or complexed with HP-*β*-CD (5, 10, 20, 30, and 40 µM) for 24 h, and then incubated with hydrogen peroxide (700 µM) for 1 h [49]. The effects on cell cultures were then evaluated by lactic dehydrogenase (LDH) release using a suitable Pierce LDH cytotoxicity assay kit. LDH activity was spectrophotometrically measured in the culture medium at *λ* = 680/490 nm by analyzing nicotinamide adenine dinucleotide (NADH) reduction during pyruvate–lactate transformation. The amount of LDH released was calculated as a percentage of the total amount.

### 2.18. Permeation Experiments through Excised Bovine Nasal Mucosa

Bovine nasal mucosa was taken from healthy animals (12–18 months of age) immediately after slaughtering and poured before use into a pH 7.0 phosphate buffer solution (PBS) containing heparin. Mucosa samples (area available for diffusion equal to 0.75 cm^2^) were mounted on Franz-type diffusion cells (LGA, Berkeley, CA, USA). The receptor of cells was filled with 4.5 mL of PBS/ethanol solution (60/40, *v*/*v*) for ensuring pseudo-sink conditions. The system was equilibrated at 37.0 ± 0.5 °C for 15 min before introducing the formulations. Aliquots of 200 µL of free and complexed IDE (30 µM), or IDE at the same dose in the presence of excess amounts of HP-β-CD (5% and 10%) in PBS were placed in the donor compartment. Samples of the receiving solution were withdrawn at different times during the experimental period (8 h) and replaced with the same amounts of fresh receptor phase previously heated at 37 ± 0.5 °C. The integrity of mucosa was assayed before and after the experiments by measuring transepithelial electrical resistance (TEER), which turned out to be 41 ± 15 Ω cm^2^. At the end of the experiment, the mucosal surface was washed with water to remove any remaining formulation, then IDE accumulated in the tissue was recovered by comminuting the nasal mucosa with a surgical blade and homogenizing in 5 mL of water with Ultra-Turrax 1 IKA (IKA1Werke GmbH & Co. KG, Staufen, Germany) for 5 min. Five milliliters of methanol was then added, and homogenization continued for 1 min. After centrifugation of the organic mixtures, the supernatants were collected and the solvent was removed under reduced pressure, yielding residues which were added to 2 mL of methanol to solubilize IDE. After filtration (0.2 µm Millipore Filter), the solutions were injected to HPLC to determine IDE. The recovery percentage of IDE was of 97% (*w*/*w*). The number of replicates was six.

### 2.19. Statistical Analysis

Data are presented as means ± standard deviation (SD). One-way ANOVA testing was carried out to evaluate statistical significance. A Bonferroni *t*-test analysis was used to validate the ANOVA test, and *p* < 0.05 was considered as statistically significant.

## 3. Results and Discussion

The IDE/ HP-*β*-CD inclusion complex was deeply characterized both in the solid state and in solution.

### 3.1. Solid-State Results

#### 3.1.1. Scanning Electronic Microscopy Analysis

**Figure 2:**
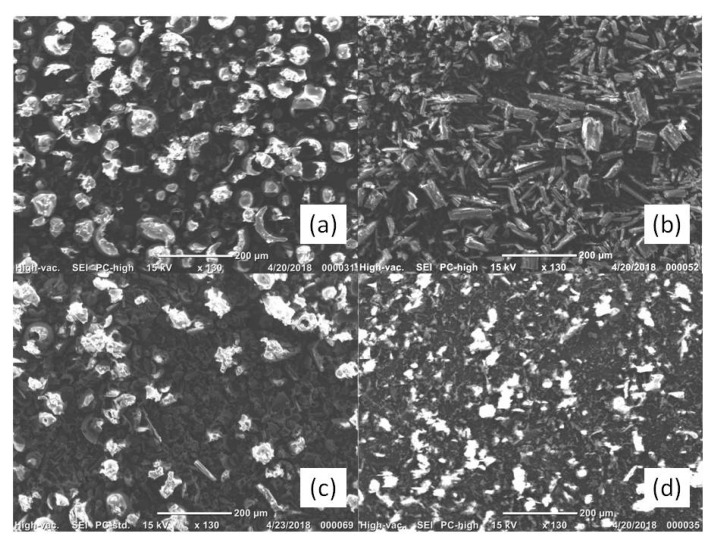
Scanning electron microscope (SEM) images of 2-hydroxypropyl-*β*-cyclodextrin (HP-*β*-CD) (**a**), idebenone (IDE) (**b**), IDE + HP-*β*-CD physical mixture (**c**), and IDE/HP-*β*-CD inclusion complex (**d**).

SEM was used to evaluate morphological changes of the pure drug and HP-*β*-CD as a result of complexation. The SEM images of pure components, IDE + HP-*β*-CD physical mixture (1:2 molar ratio) and the solid IDE/HP-*β*-CD inclusion complex at the same molar ratio, are reported in Figure 2.

The SEM photographs put into evidence the morphological differences between the single components, physical mixture, and inclusion complex. HP-*β*-CD (Figure 2a) was observed to have an almost circular form, irregular with a little wrinkled surface. Pure IDE (Figure 2b) appeared as needle-like crystals. Both HP-*β*-CD particles and IDE were still evident in the SEM image of the physical mixture (Figure 2c). On the contrary, drastic changes in morphology and shape of particles of the inclusion complex were revealed (Figure 2d). In particular, the original morphology of the two pure compounds was lost, while an amorphous powder with reduced sizes and no differentiation of the individual components was observed. These modifications can be considered as proof that a new solid phase existed [50] and could have been reason for the quick dissolution rate observed for the inclusion complex.

#### 3.1.2. FTIR-ATR Studies

The FTIR-ATR spectrum in the high MIR range of IDE [30] exhibited, as primary features, a peak at ~3570 cm^−1^, corresponding to the O–H stretching vibration, and a double-peak centered at ~2919 cm^−1^ and ~2845 cm^−1^, ascribed to the C–H stretching vibrational mode. As far as the low MIR spectral range was concerned, a double-peak at ~1645 cm^−1^ and ~1608 cm^−1^ was observed, indicating the carbonyl C=O (~1645 cm^−1^) and ring C=C (~1608 cm^−1^) stretching vibrations. Going on, the 1500−1000 cm^−1^ spectral range was mainly associated with the C–C and C–O stretching vibrations. Finally, in the 900−600 cm^−1^ region, the bands corresponding to out-of-plane bending of aromatic C–H bonds were detected.

HP-*β*-CD showed [51] a broad band between the 3700 and 3000 cm^−1^ region, reflecting the stretching of free and bound O–H groups. It was convoluted with another band extending from 3000 to 2700 cm^−1^, describing the C–H stretching vibrational mode. At ~1637 cm^−1^ the *δ*-HOH bending vibration of H_2_O molecules attached to CD was detected. The spectral features centered at ~1157 cm^−1^ and ~1020 cm^−1^ were respectively due to C–H and C–O stretching modes.

**Figure 3:**
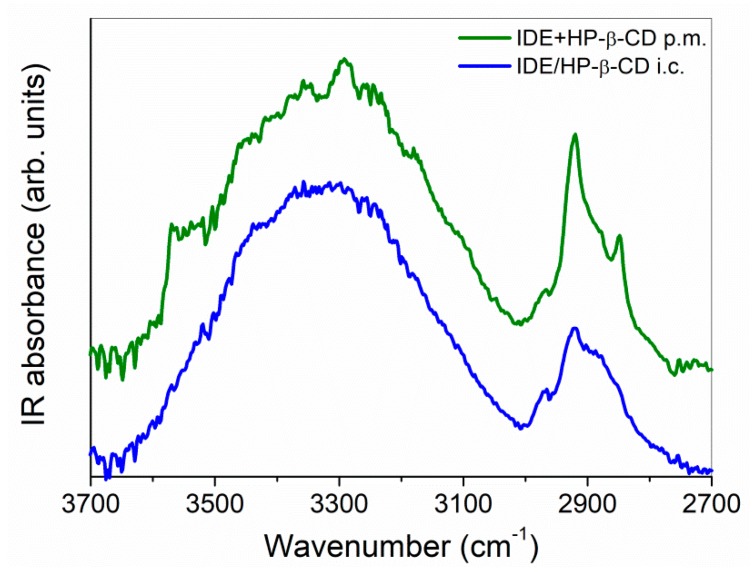
Fourier-transform infrared spectroscopy in attenuated total reflectance geometry (FTIR-ATR) spectra, in the 3700–2700 cm^−1^ mid-infrared spectral range, of IDE + HP-*β*-CD physical mixture (p.m., green line) and IDE/HP-*β*-CD inclusion complex (i.c., blue line), at T = 300 K as an example. IR: infrared.

As widely recognized [52,53,54,55], the investigation through FTIR-ATR technique of the IR-active O-H stretching mode results particularly informative of the intra- and intermolecular modes of these specific functional groups, whose oscillator forces are sensitive, in turn, to the interactions with a solute or in general with their surroundings. It furnishes an indirect picture of the hydrogen bond (HB) network, since changes in the bond strength and relative populations of differently H-bonded molecules correspond to variations in its shape and position [56]. In our case, the changes observed in the O–H stretching vibration passing from the physical mixture to inclusion complex (green line and blue line, respectively, in Figure 3) could allow probing the alteration of the H-bonded environments upon complexation.

In particular, we observed the disappearance, in the FTIR-ATR spectrum of the inclusion complex, of the high-frequency contribution well evident in the experimental profile of the physical mixture, associated to the stretching mode of “free” OH groups of pure IDE. At the same time, the O–H band of the inclusion complex appeared enlarged with respect to that of the physical mixture and slightly shifted to higher wavenumbers. These occurrences testified, on one side, the involvement of OH groups of IDE in new host–guest interactions through the formation of a hydrogen bond. Furthermore, the H-bond network appeared globally weakened upon complexation.

**Figure 4:**
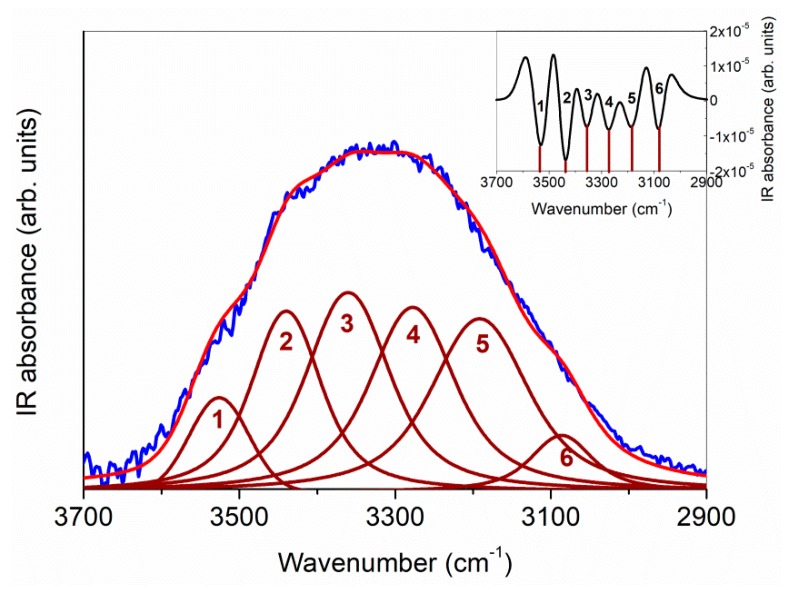
A typical example of best fit results for FTIR-ATR spectrum, in the 3700–2900 cm^−1^ range, of the IDE/HP-*β*-CD 1:2 inclusion complex (blue line), at T = 290 K as an example. In the inset, the corresponding diagram of the second-derivative computation of the experimental spectrum is shown, clearly evidencing the existence of six minima. See text for details.

A widely recognized approach to satisfactorily account for, in a quantitative way, such modifications of the H-bond network in cyclodextrin-based systems [54,57,58] is to perform, after a proper subtraction of the overlapping C–H vibrational mode [59], a curve-fitting procedure of the O–H stretching band into six components accounting for interstitial (*ω*_3_ ~3360 cm^−1^, *ω*_6_ ~3084 cm^−1^) and intracavity (*ω*_1_ ~3525 cm^−1^) water molecules, primary (*ω*_2_ ~3439 cm^−1^) and secondary (ω_5_ ~3191 cm^−1^) OH groups of HP-*β*-CD, and OH groups of IDE (*ω*_4_ ~3277 cm^−1^). The analysis is supported by the calculation of the second derivative of the IR spectra, furnishing a first indication of the number of sub-bands and their center-frequencies. Figure 4 reports a typical example of best fitting results, at T = 290 K as an example.

**Figure 5:**
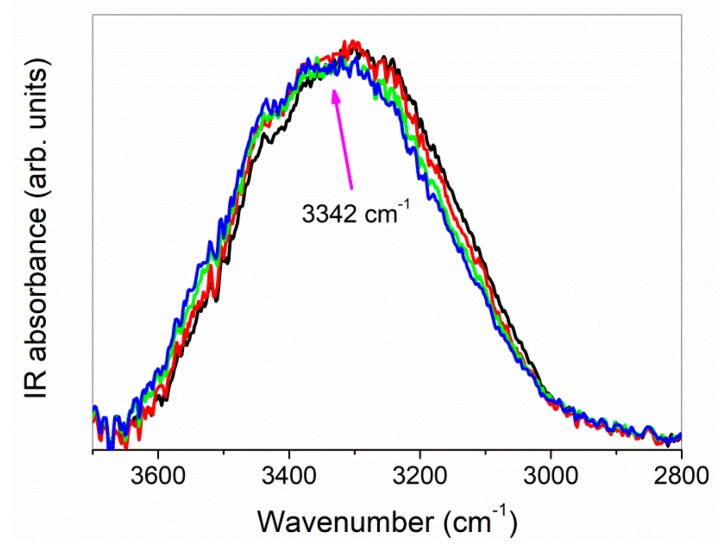
Experimental FTIR-ATR spectra for the IDE/HP-*β*-CD 1:2 inclusion complex, in the O–H stretching region, plotted vs. T at 250 K (black line), 280 K (red line), 300 K (green line), and 340 K (blue line) as examples. The isosbestic point is centered at ~3342 cm^−1^.

The fitting procedure applied to the FTIR-ATR O–H stretching spectra could be used to estimate the average strength of the H-bond network developed upon complexation. In order to do so, we verified, first of all, the existence of an isosbestic point for the O–H stretching band. In Figure 5, we report the evolution, as a function of temperature, of the FTIR-ATR experimental spectra in the O–H stretching region.

**Figure 6:**
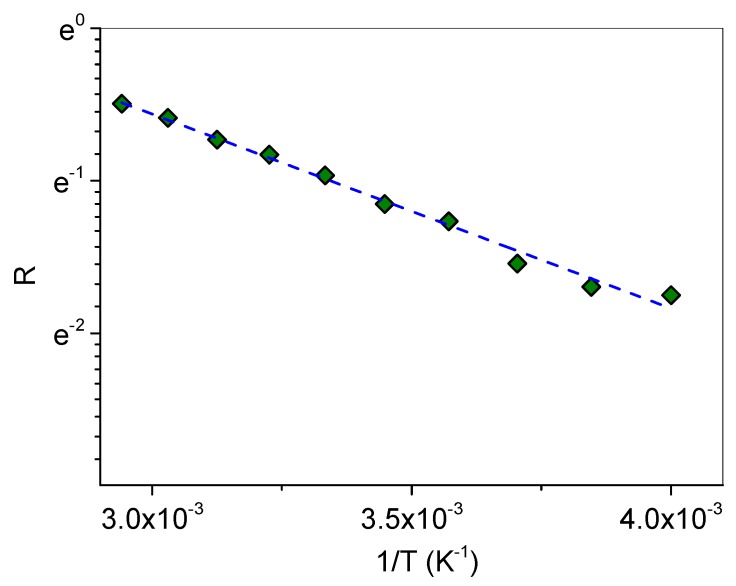
Semi-log plot of the temperature dependence of *R*.

As already reported [60], an isosbestic point represents a spectroscopic indication of a reversible chemical reaction among two states. If two *transient* structural environments (having a lifetime on the order of picoseconds), whose respective spectral features are overlapping, suffer a kinetic process under a temperature change, their spectra will cross at a single critical point. In such a case, the spectrum can be considered as the sum of two independent contributions. For our system, we considered a first class of non-H-bonded (or at least slightly H-bonded) oscillators, described by the sub-bands *ω*_1_ and *ω*_2_, whose populations are accounted by the sum of the percentage intensities *I*_1_ + *I*_2_ as obtained from the best fit, and a second class due to H-bonded (HB) species, represented by the sub-bands *ω*_i_ (i = 3, …, 6), whose population is expressed by *I*_3_ + *I*_4_ + *I*_5_ + *I*_6_. In the framework of the two-state model [61], the van ’t Hoff plot of the ratio *R* = *I*_1_ + *I*_2_/*I*_3_ + *I*_4_ + *I*_5_ + *I*_6_, as reported in Figure 6, furnished the average enthalpy Δ*H*_HB_ of the HB scheme upon inclusion.

From the linear fit according to ln*R* = Δ*H*_HB_/*RT* + Δ*S*_HB_/*R*, a value of Δ*H*_HB_ = 10596 ± 390 J·mol^−1^ was obtained.

Complexation was proven to involve also CH functional groups, as evidenced by the relevant changes in the C–H stretching region, through the strong attenuation, passing from the physical mixture to inclusion complex, of the peaks at ~2922 cm^−1^ and ~2848 cm^−1^, respectively assigned to the C–H mode of the groups belonging to the ring and to the alkyl chain.

**Figure 7:**
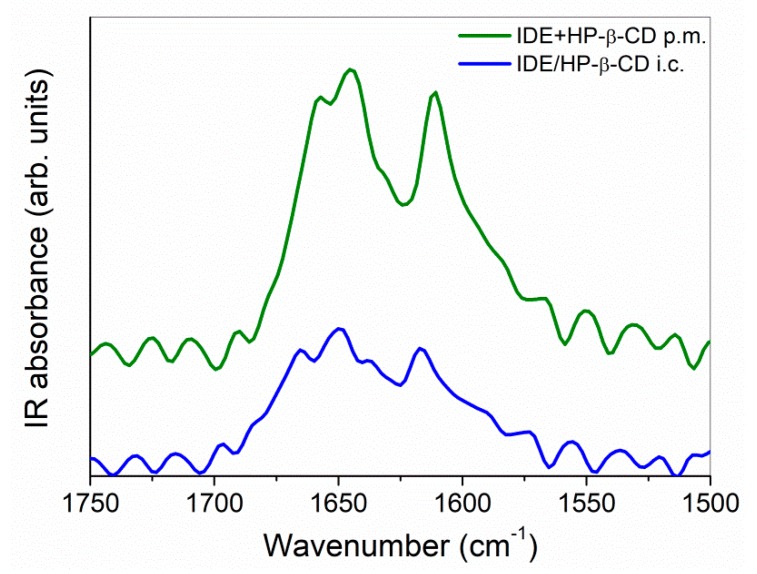
FTIR-ATR spectra, in the 1750–1500 cm^−1^ mid-infrared spectral range, for the IDE + HP-*β*-CD physical mixture (p.m., green line) and IDE/HP-*β*-CD inclusion complex (i.c., blue line), at T = 300 K as an example.

Also, the changes revealed in the C=O and C=C stretching region, passing from the physical mixture to inclusion complex (Figure 7), are very important in view of the elucidation of the molecular state of the drug.

A broadening of the band was well evident, indicating a restriction of these groups due to the inclusion within the cavity of HP-*β*-CD. More interesting, a shift toward the high wavenumber region was in particular observed for the maxima of both the C=O (from ~1645 cm^−1^ to ~1650 cm^−1^) and the C=C (from ~1608 cm^−1^ to ~1616 cm^−1^) stretching modes. This shift, according to previous literature on similar systems [62], could be ascribed to a change in polarity of the environment around the C=O and C=C groups upon complexation, which caused a reinforcement of the dipole moment associated to them, shifting the corresponding stretching vibration frequencies toward higher values. This weakened environment was, in turn, associated with the breakdown of intermolecular hydrogen bonds of the guest and the subsequent formation of a monomeric dispersion of the drug because of the establishment of host–guest interactions, thus confirming the inclusion process.

Nevertheless, a deeper interpretation of these changes could become less reliable due to the overlapping of these contributions with that coming from the *δ*-HOH bending vibration of crystallization water molecules of the macrocycle, as clearly detected at ~1637 cm^−1^ in the FTIR-ATR spectrum of HP-*β*-CD. In this case, the use of a complementary methodology [63], such as Raman spectroscopy, becomes mandatory for a complete vibrational analysis.

#### 3.1.3. FT-NIR Studies

**Figure 8:**
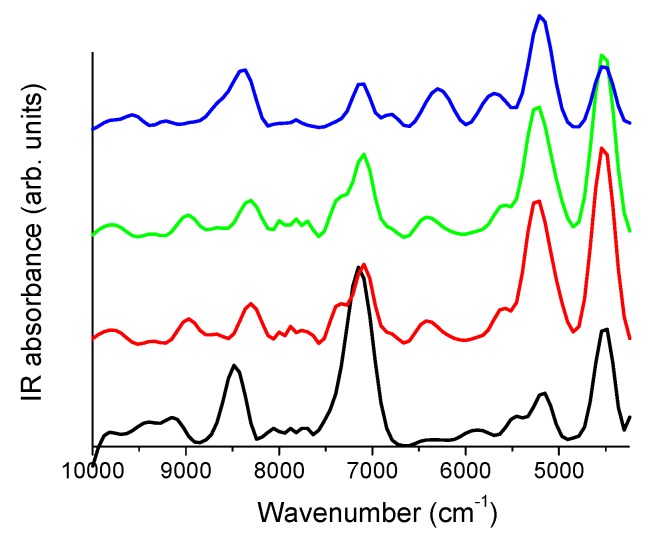
Near-infrared (NIR) spectra, in the 10,000–4250 cm^−1^ spectral range, of IDE (black line), HP-*β*-CD (red line), IDE + HP-*β*-CD 1:2 physical mixture (green line), and IDE/HP-*β*-CD 1:2 inclusion complex (blue line).

FT-NIR spectra collected for the IDE, HP-*β*-CD, IDE + HP-*β*-CD physical mixture (1:2 molar ratio), and IDE/HP-*β*-CD inclusion complex (1:2 molar ratio) are reported in Figure 8.

The spectrum of IDE showed features between ~9500 and ~9000 cm^−1^, as well as a broad band centered at ~8500 cm^−1^, ascribed to the second overtone of C–H functional groups. Going on, the main band centered at ~7150 cm^−1^ was principally attributed to the first overtone of O–H vibrations. The bands observed between ~6500 cm^−1^ and ~5500 cm^−1^ reflected the first overtone of C–H groups, whereas, at ~5170 cm^−1^, the contribution of the first overtone of C=O groups was evident. Finally, the intense peak at ~4512 cm^−1^ was mainly associated with the C–C combination band.

As far as the FT-NIR spectrum of HP-*β*-CD is concerned, the second overtone of C–H groups gave rise to the bands observed at ~8975 cm^−1^ and ~8295 cm^−1^. The first overtone of primary and secondary hydroxyl groups of the macrocycle was responsible for the broad and complex band observed between ~7565 cm^−1^ and ~6845 cm^−1^. The shoulder observed at ~6775 cm^−1^ was ascribed to the second overtone of crystallization water molecules. The two bands at ~6390 cm^−1^ and ~5610 cm^−1^ corresponded to the first overtone of the C–H groups, whereas the intense peak at ~5242 cm^−1^ was associated with the first overtone of crystallization water molecules, other than the first overtone of C=O groups. Finally, the C–C combination band was evident at ~4502 cm^−1^.

The spectrum of the physical mixture showed a plain superimposition of the drug and CD spectra, indicating that no interaction occurred between IDE and HP-*β*-CD [64]. On the other side, the spectrum of the inclusion complex showed relevant spectral modifications. The bands associated with the second overtone of the C–H groups exhibited shifts and changes in the relative intensity, indicating changes in the vibration energies of IDE, thus confirming the formation of intermolecular bonds with HP-*β*-CD. At the same time, the disappearance, in the NIR spectrum of inclusion complex, of the high-frequency contribution at ~7352 cm^−1^ to the first overtone of the O–H groups, well detectable in the spectrum of the physical mixture, testified intermolecular host–guest interactions occurring via hydrogen bond. The shape of the bands of the first overtone of C–H groups appeared strongly affected by complexation, as well as the band associated with the first overtone of H_2_O molecule and C=O group shifts in frequency. Finally, the combination C–C band appeared, in the inclusion complex, reduced in intensity and strongly enlarged with respect to the physical mixture, suggesting a hindering of the corresponding vibration because of the entrapment of the C–C functional group inside the hydrophobic cavity of CD.

#### 3.1.4. Raman Studies

**Figure 9:**
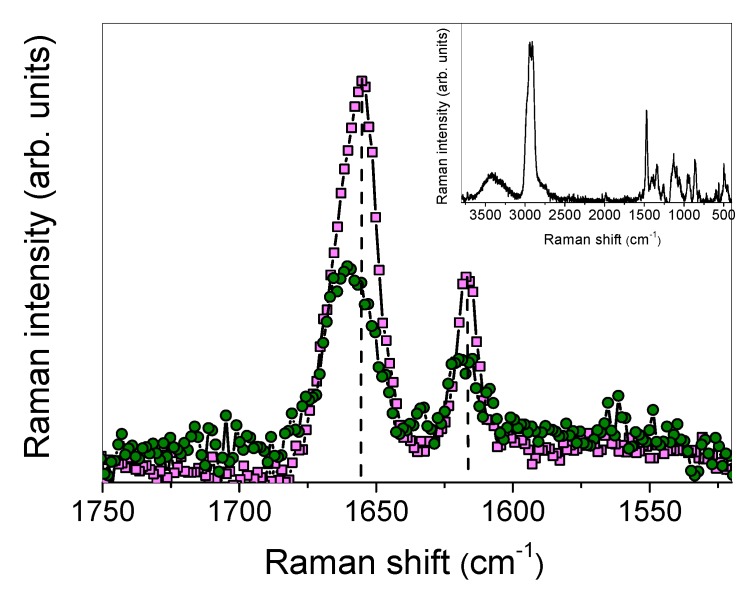
Raman spectra, in the 1750–1520 cm^−1^ range, of IDE (pink squares) and IDE/HP-*β*-CD 1:2 inclusion complex (green circles). In the inset, the Raman spectrum of HP-*β*-CD in the 1750–1520 cm^−1^ range is also reported.

As shown in Figure 9, two intense and well-resolved peaks were observed in the Raman spectrum of IDE, centered at ~1655 cm^−1^ and ~1616 cm^−1^, in a range lacking HP-*β*-CD bands (see inset of Figure 9). Hence, they could be used as markers for complexation [65]. By comparison with structurally related compounds [66], they could be respectively ascribed to C=O and C=C stretching vibrations of the ring.

When looking at the Raman spectrum of the inclusion complex, the intensity of the peaks corresponding to C=O and C=C stretching modes was reduced with respect to free IDE, and a shift toward higher wavenumbers was revealed, of ~6 cm^−1^ and ~3 cm^−1^, respectively. A broadening of these bands was, at the same time, observed in the Raman spectrum of the complex.

These features indicate, in agreement with the FTIR-ATR data, that IDE in co-precipitated systems suffered an environment significantly different than that of the non-complexed state. In particular, the high-wavenumber shift of C=O and C=C vibrations testified a reinforcement of the dipole moment associated with these bonds, reasonably caused by a less strong association where one IDE molecule interacted, through intermolecular hydrogen bond, with two HP-*β*-CD cavities. Also, the presence of an unconventional H-bond between C–H groups of the IDE ring and OH groups of HP-*β*-CD cannot be a priori excluded, as suggested by the vibrational modifications of C=C groups.

The interaction between C=O and C=C groups with HP-*β*-CD was also confirmed by the increase in bandwidths, which implies the decrease in vibrational relaxation times revealing a hindering of the vibrations themselves, due to the restriction of the functional groups in the cyclodextrin cavity. These occurrences could be considered as a confirmation of the existence of the complex in the solid state, whose complexation geometry is later clarified.

### 3.2. In Solution Studies

#### 3.2.1. Phase Solubility Studies

**Figure 10:**
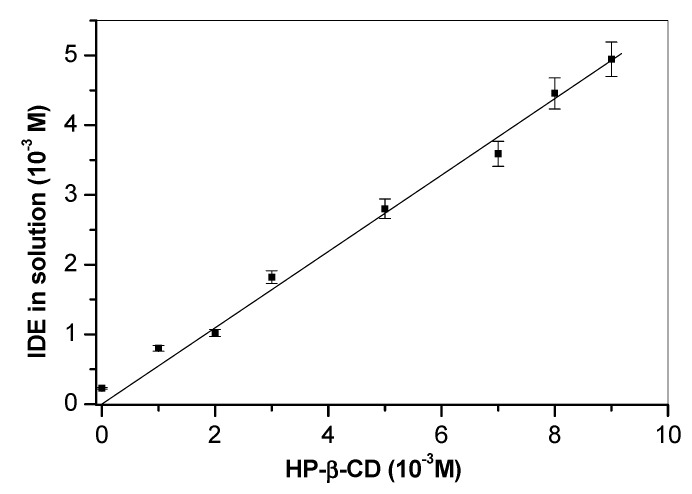
Phase-solubility diagrams of IDE upon increasing the concentration of HP-*β*-CD in water at 25.0 ± 0.5 °C.

Phase-solubility studies were carried out to evaluate the host–guest interactions between the drug and HP-*β*-CD. The resulting isotherm displayed a linear increase in IDE water solubility with the increasing concentration of the macrocycle, indicating a favorable interaction between host and guest, giving rise to the formation of a soluble complex. An *A*_L_ type curve was obtained with a slope below 1 (Figure 10), showing the formation in the aqueous solution of a complex in 1:1 molar ratio with a stability constant value (*K*_c_), calculated according to Higuchi and Connors [35] equation, of 6031 M^−1^ [31].

A very high increase of solubility in water of the inclusion complex with respect to free IDE was observed, together with a quick dissolution rate (100% dissolution within 15 min in water) [31].

#### 3.2.2. UV–Vis Titration Experiments

UV–Vis titration experiments were also performed to determine the stoichiometry of the obtained IDE/HP-*β*-CD inclusion complex, as well as to further evaluate its association constant.

The analysis and fitting of the absorption data at all wavelengths were performed by HypSpec software. Different stoichiometries were studied (1:1, 1:2, and 2:1 IDE:HP-*β*-CD). The fitting method was based on the analysis of residual distribution in titration data fitting, considered the most reliable for establishing a proper binding model [67,68]. A systematic trend was not shown by the residuals of the 1:1, suggesting that no other stoichiometries of the complex were formed under these experimental conditions. The calculated *K*_c_ resulted equal to 6025 M^−1^, which was in excellent correlation with the *K_c_* value obtained from the phase-solubility diagram according to the Higuchi and Connors equation [35].

#### 3.2.3. Nuclear Magnetic Resonance Studies

**Figure 11:**
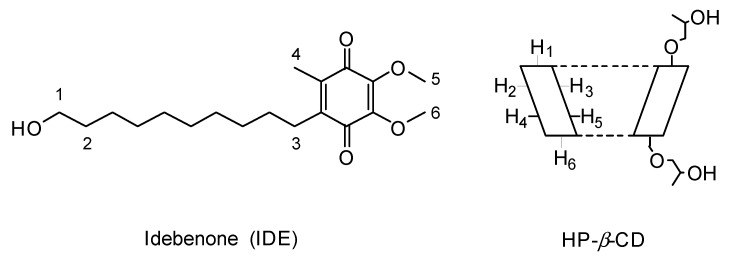
Structure of IDE and schematic structure of HP-*β*-CD. The numeration of IDE and HP-*β*-CD group is arbitrary for simplicity.

**Figure 12:**
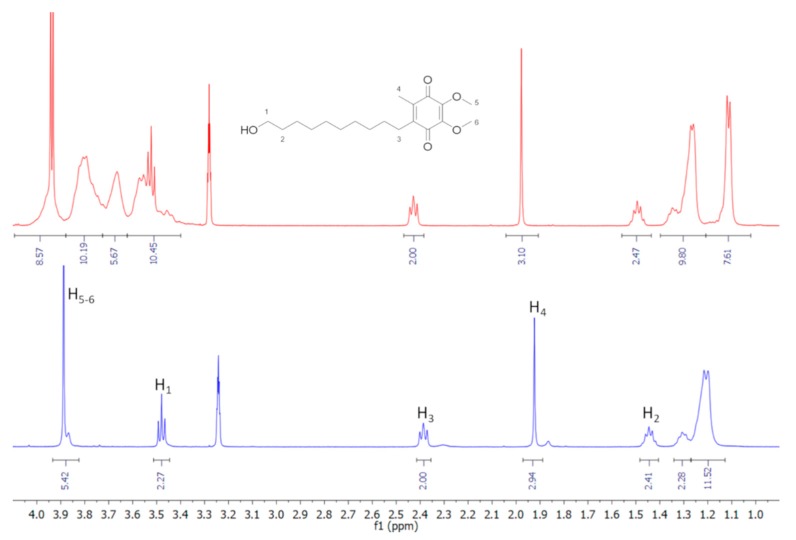
Stacked portions of ^1^H-NMR spectra relative to free IDE (bottom) and IDE/HP-*β*-CD complex (top).

Nuclear magnetic resonance (NMR) spectroscopy is a useful technique employed to study the interaction between ligand and carrier molecule [69], since the chemical and electronic environments of the protons are affected during CD complexation, and these changes are reflected in the shifts of corresponding groups (chemical-induced shift, CIS). Unfortunately, as with most of the substituted CDs, the HP-*β*-CD derivative can be considered as a statistical mixture of different stereoisomers, due to its chemical modifications. Therefore, it has broad unresolved peaks, making it almost impossible to follow the chemical shifts of its internal H_3_ and H_5_ protons, normally used to prove the formation of inclusion complexes. Thus, this last one was deduced from the chemical shift changes of IDE. In Figure 11, the structure of IDE and a schematic representation of HP-*β*-CD are shown. The staked portion of the ^1^H NMR spectra of the IDE and IDE/HP-*β*-CD inclusion complex are shown in Figure 12, and the chemical shifts are tabulated in Table 1. The full spectra of the IDE, HP-*β*-CD, and IDE/HP-*β*-CD inclusion complex are shown in the Supplementary Materials (Figures S2–S4).

The inclusion of IDE into the HP-*β*-CD cavity was confirmed by changes in the chemical shifts of the guest protons in comparison with the chemical shift of the same protons in the free compounds. Generally, in the presence of HP-*β*-CD, the guest protons were downfield shifted (Table 1). A downfield displacement of the IDE protons indicated that they were close to an electronegative atom, like oxygen.

**Figure 13:**
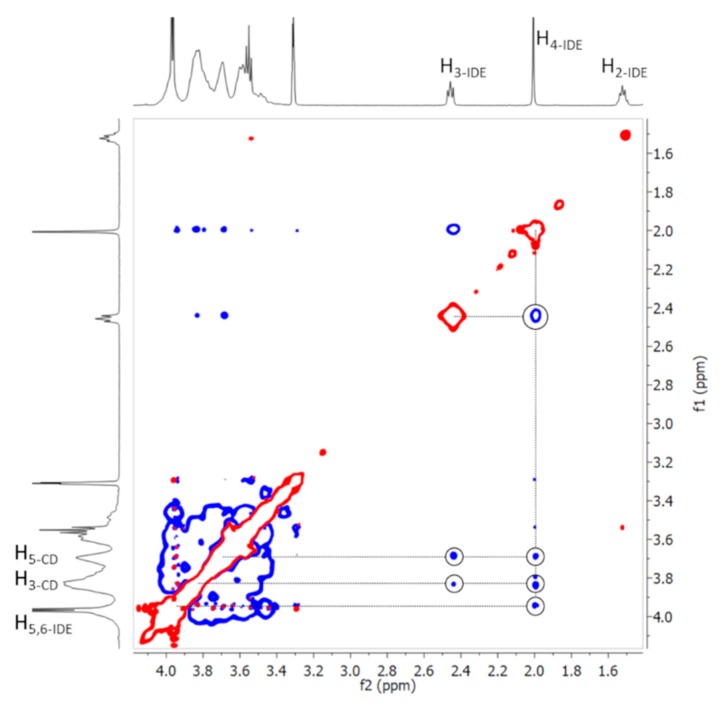
Expansion of two-dimensional (2D) rotating frame Overhauser effect spectroscopy (ROESY) plot of IDE/HP-*β*-CD complex in D_2_O/MeOD, showing the NOEs (blue) of interest.

The formation of the inclusion complexes was further confirmed from ROESY (rotating frame Overhauser effect spectroscopy) experiments (Figure 13). In this type of experiment, inter- and intramolecular interactions can be observed. If two protons from different compounds are in spatial vicinity within 3–5 Å, an NOE cross-peak is observed in the 2D ROESY spectrum (see Supplementary Materials, Figure S5).

The expanded ROESY spectrum of the IDE/HP-*β*-CD inclusion complex (Figure 13) showed NOE cross-peaks for the H_3_ and H_4_ of IDE protons and the internal H_3_ and H_5_ of HP-*β*-CD, confirming once again the formation of the inclusion complex. Intramolecular cross-peaks for IDE were present between H_3_/H_4_, H_3_/H_aliphatics_, and H_4_/H_5,6_. Worthy of note, the H_2_ of IDE did not have any NOE cross-peaks with internal protons of the CD, suggesting that the geometry of the host/guest complex was the one with the quinone part of the molecule inside the CD ring.

**Figure 14:**
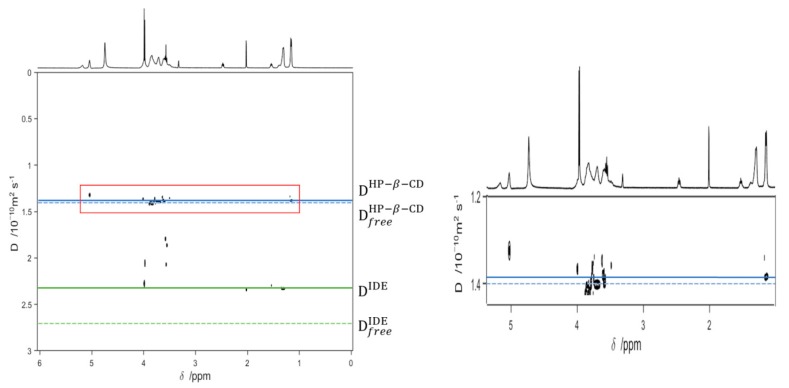
On the left: 2D diffusion ordered spectroscopy (DOSY)-NMR measurements on the 14 mM aqueous methanol solutions (D_2_O/CD_3_OD 1:1) of the IDE, HP-*β*-CD, and IDE/HP-*β*-CD inclusion complex. The horizontal axis represents the chemical shifts; the vertical axis represents the diffusion coefficients. The black spots are the resonances of the aqueous solution of the inclusion complex spread in the second dimension according to their measured diffusion coefficient. On the right: enlarged representation of the red box.

Finally, to further demonstrate the inclusion of IDE, we performed a series of DOSY experiments on free IDE, free HP-*β*-CD, and IDE/HP-*β*-CD complex (Figure 14). The green and solid blue lines represent the diffusion coefficient measured for IDE and HP-*β*-CD in the complex, respectively. The corresponding dashed lines represent the diffusion coefficients of free IDE and free HP-*β*-CD. The extent to which the solid lines in Figure 14 were displaced from their corresponding dashed lines provided the basis for the quantitative estimation of the complex formation constant.

The results pointed out that the diffusion coefficient for IDE decreased from 2.69 × 10^−10^ m^2^/s to 2.30 × 10^−10^ m^2^/s, and this was indicative of a complexation inside the CD cavity. In the same way, the diffusion coefficient of HP-*β*-CD also diminished, but slightly.

### 3.3. Molecular Modeling Studies

**Figure 15:**
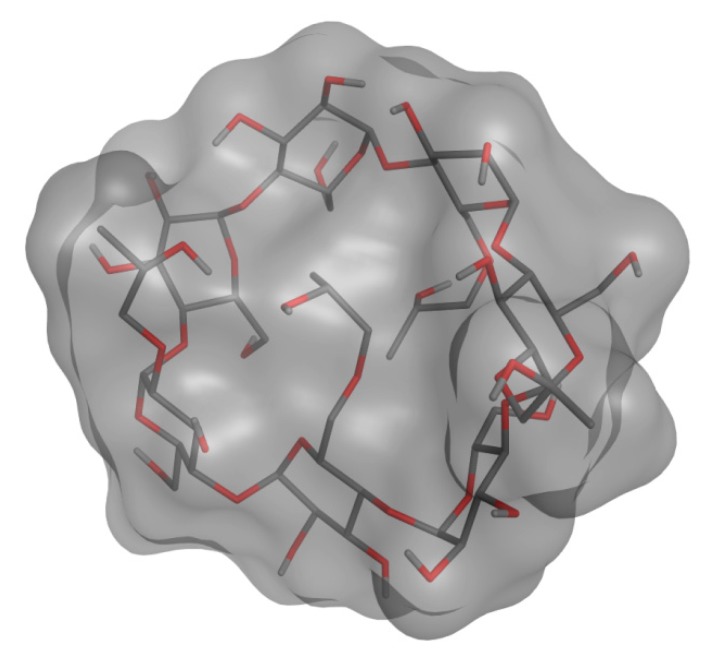
Structure of the closed conformation of the HP-*β*-CD model with two substitutions on the wide rim and other two substitutions on the narrow rim.

In order to investigate the IDE/HP-*β*-CD host–guest interactions, we started the molecular modeling study with a molecular dynamics (MD) simulation. Two different MD simulations were carried out using different HP-*β*-CD. In the first one, four oxygens on the C_2_ position (residues 1, 3, 5, and 7) were substituted with the 2-hydroxypropyl group; in the second one, the four oxygens on the C_6_ position (residues 1, 3, 5, and 7) were substituted with the same group. A model with two substitutions on the wide rim and other two substitutions on the narrow rim was also studied but not reported because, after 15 ns and for the entire simulation time, the entrance to the CD cavity resulted obstructed by the 2-hydroxypropyl groups, due to the formation of intramolecular hydrogen bonding (Figure 15).

**Figure 16:**
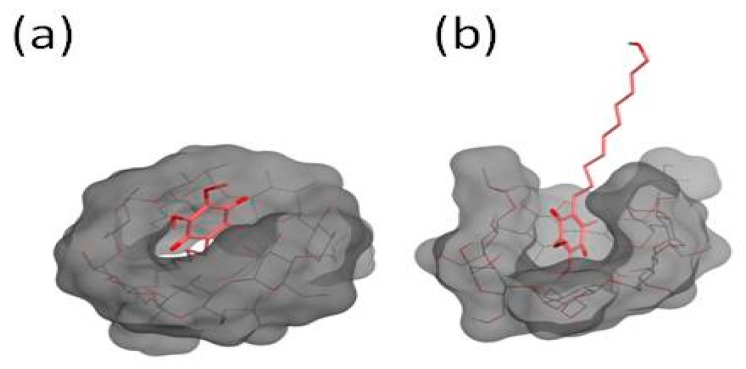
Optimized structure of IDE in the HP-*β*-CD cavity.

**Figure 17:**
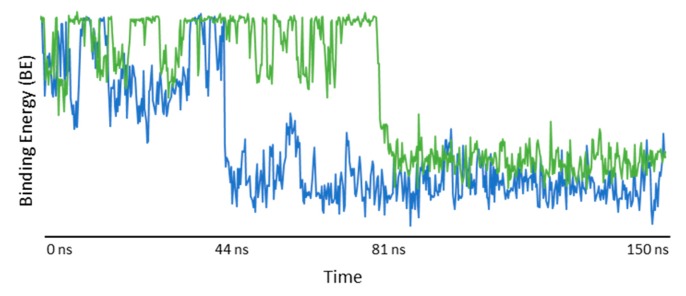
Poisson–Boltzmann binding energy (BE) during the entire time of the molecular dynamics (MD) simulation. Blue line: BE of the model with four 2-hydroxypropyl groups to the O_6_ of the CD. Green line: BE of the model with four 2-hydroxypropyl groups to the O_2_ of the CD.

The analysis of the first simulation (Model (a) in Figure 16) showed that, after 81 ns, the IDE molecule went inside the hydrophobic cavity from the narrow rim (Figure 17). Once inside the host molecule, IDE established interactions that stabilized the complex, and, for the entire simulation time (150 ns), it remained inside the CD, maintaining the same orientation. In the second simulation (Model (b) in Figure 16), IDE went inside the HP-*β*-CD after 44 ns (Figure 17) from the wide rim, and established interactions that stabilized the complex for the entire simulation time (150 ns).

In both MD simulations, it is interesting to notice that IDE was included inside the hydrophobic ring from the quinone part of the molecule, with the alkyl chain pointing out of the CD, in agreement with the NMR study, and it was always aligned with the tethered 2-hydroxypropyl groups. Starting from the minimized structure of the complex, obtained from the last 5 ns of the MD simulation, we performed an MM/PBSA calculation to get the binding energy and, consequently, the association constant. The optimized structures of the IDE/HP-*β*-CD complex, reported in Figure 16, were consistent with the complex geometry deduced by the ROESY experiments. The binding energies of the models (a) and (b) were −5.75 and −5.31 kcal/mol, respectively, in good agreement with the experimental value (*K_c_* = 6025 M^−1^; Δ*G* = −5.15 kcal/mol).

### 3.4. In Vitro/Ex Vivo Biological Studies

#### 3.4.1. Cytotoxicity Studies

Human glioblastoma astrocytoma (U373) cell culture tolerability toward free and complexed IDE was assayed by performing an MTT test.

We evaluated five concentrations of IDE as free drug or complexed with HP-*β*-CD (5, 10, 20, 30, and 40 μM) for 24, 48, and 72 h. All samples were prepared in water; thus, solutions were obtained in all cases, except for free IDE at 30 and 40 μM, which gave rise to suspensions, as these concentrations were higher than its water solubility (about 22 μM).

Cell viability was unaffected by free IDE until the 20 μM concentration, but higher doses produced a loss of cell viability, probably due to the presence of solid IDE that could interfere with the vital cycle of the cells or, as reported by other authors [70,71], because of the cytotoxic activity showed by IDE at concentrations higher than 25 μM.

**Figure 18:**
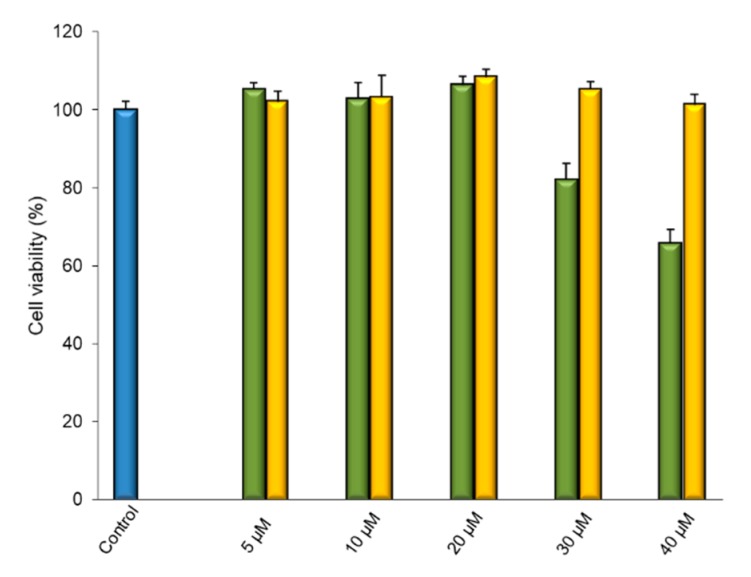
Effects on U373 cell viability of 24-h treatment with different concentrations of free (green bar) and complexed IDE (yellow bar). Cells were treated with free and complexed IDE at the indicated concentrations, and cell viability was measured using the MTT test. Results are presented as the means of three different experiments (six replicates for each point) ± standard deviation.

No influence was produced by complexed IDE on cell viability, at all assayed doses. The absence of cytotoxicity observed in this case at the highest doses of IDE could be due to the high solubility of the complex; thus, so no contact of cells with solid IDE occurred. Moreover, it must be considered that only the free drug, and not the complexed form [72], was able to interact with the cells. Now, due to the high stability constant of the inclusion complex (about 6000 M^−1^), the amount of free IDE in equilibrium with the complex, available to interact with the cells, was lower than the effective applied dose, and probably below the minimum dose of IDE that shows cytotoxic activity. In Figure 18, we present the data relative to the 24-h treatment, as an example. Similar results were obtained at all times.

#### 3.4.2. Evaluation of Antioxidant Activity

CDs show great ability to increase the antioxidant activity of different drugs. Generally, this positive effect is ascribed to the significant increase in water solubility of drugs, following their complexation [73,74], which produces a high availability of the drug at the site of action. CDs could also act as “secondary antioxidants”, improving the ability of traditional antioxidants to prevent enzymatic browning [33]. *β*-CD was proven to potentiate the antioxidant activity of the natural product rutin because of the penetration of the *o*-dihydroxyphenol moiety of the polyphenols within the CD cavity, which probably stabilized the semiquinone-radical oxidation product [75]. Roy et al. [76] demonstrated that the complexation of the quinone ring of (−)-epicatechin-gallate and (−)-epigallocatechin-gallate into *β*-CD prevented protein oxidation and restricted the formation of higher protein oligomers, increasing the antioxidant efficiency of the two actives. Inclusion complexes of daidzein with *β*-CD, methyl-*β*-CD, and HP-*β*-CD showed better antioxidant activity with respect to the free drug, with the daidzein-HP-*β*-CD inclusion complex being the most effective [77].

Based on the aforementioned literature, we evaluated the influence of HP-*β*-CD on the antioxidant activity of IDE by in vitro studies on U373 cell cultures. Oxidative stress was induced in cells by hydrogen peroxide (H_2_O_2_). As the H_2_O_2_-induced cellular necrosis of tissues is associated with the release of lactic dehydrogenase (LDH), the integrity of the cytoplasm membrane can be tested by measuring the percentage of LDH released in the medium. Before the oxidative stress, cells were pretreated with free and complexed IDE at drug concentrations ranging between 5 and 40 μM, to evaluate its protective effect against the H_2_O_2_-induced cell damage.

**Figure 19:**
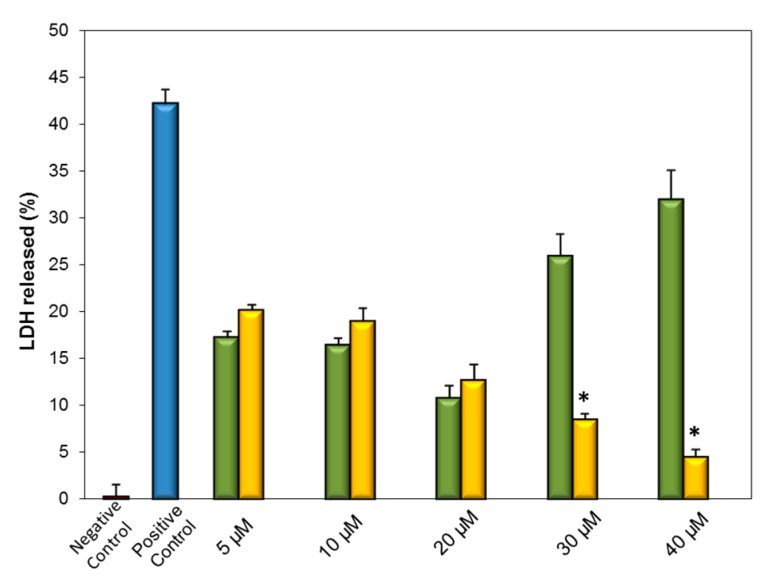
Protective effect of different concentrations of free (green bar) and complexed (yellow bar) IDE on U373 cells against hydrogen peroxide damage. Cells were treated with free and complexed IDE for 24 h; then, they were incubated with hydrogen peroxide for 1 h. LDH release was assessed as described in the Section 2. Results are presented as the means of three different experiments (six replicates for each point) ± standard deviation; * different from free IDE (*p* < 0.05).

Addition of H_2_O_2_ to U373 cells caused a strong release of LDH (about 40%) with respect to control cells (Figure 19), which showed 100% vitality. As expected, free HP-*β*-CD showed no significant advantage in terms of in vitro antioxidant effect (data not shown). A different trend was observed for the free IDE and soluble IDE/HP-*β*-CD inclusion complex. Soluble free IDE (5, 10, and 20 μM) showed a notable reduction of H_2_O_2_-induced damage in a dose-dependent way. The highest doses of free IDE (30 and 40 μM) produced no protective effect but, on the contrary, a significant cytotoxic effect was observed, as expected by the previously reported tolerability test.

Cells pretreated with the IDE/HP-*β*-CD inclusion complex showed a protective effect against H_2_O_2_-induced damage, particularly at the highest concentrations, demonstrating a clear dose-dependent profile. At lower doses (5, 10, and 20 μM) free IDE was more active than the complex. As observed by Loftsson et al. [72], both CDs and their inclusion complexes are not absorbed through biomembranes. Only the free form of the drug, which is in equilibrium with the complex, is able to interact with lipophilic membranes. Furthermore, the high amount of CDs needed for complexation could reduce the permeation of included drugs [72].

On this basis, it is reasonable that the high stability constant of the IDE/HP-*β*-CD inclusion complex produced a limited release of IDE from the complex. As a consequence, the dose of released IDE that effectively interacted with U373 cells was less than the corresponding dose of free IDE, resulting in a lower protective effect. However, a high protective effect was observed when the cells were pretreated with 30 and 40 μM of complexed IDE. In particular, at 40 μM, the inclusion complex produced only 4% of LDH release (Figure 19), which can be justified as discussed in the previous section. A deeper investigation is at the present in progress in order to clarify this aspect.

#### 3.4.3. Permeation Experiments through Excised Bovine Nasal Mucosa

As demonstrated [34,78], HP-*β*-CD can increase the permeation of an included drug across biomembranes. This property was generally ascribed to the ability of CD to dissolve and deliver lipophilic drug through the aqueous exterior of the biomembrane, thus increasing the drug concentration gradient over the lipophilic membrane [79]. In addition, the ability of HP-*β*-CD to extract cholesterol from biomembranes [80] or from atherosclerotic plaque [81] was also shown. HP-*β*-CD increased the brain bioavailability of levo-dopamine (L-DOPA) administered intranasally, even if no increase of drug water solubility was observed [82]. It was also reported that HP-*β*-CD increased the flux of IDE through bovine buccal mucosa to a greater extent than the other penetration enhancers tested, thus concluding that the IDE/HP-*β*-CD complex can itself act as a penetration enhancer for buccal drug delivery of the drug [83].

**Figure 20:**
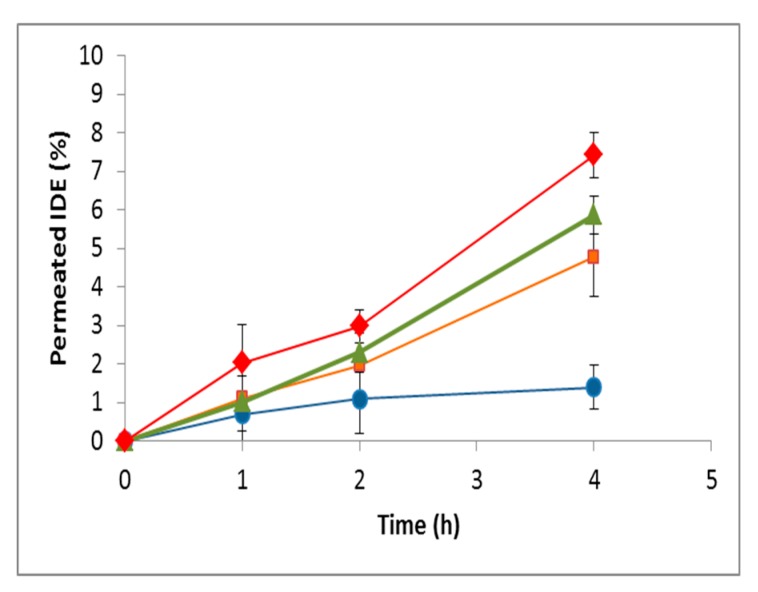
Permeation profiles across the excised bovine nasal mucosa of free IDE (blue circles) and in the presence of increasing amounts of HP-*β*-CD. Orange squares: 1:2 IDE:HP-*β*-CD inclusion complex, green triangles: 5% HP-*β*-CD, red diamond: 10% HP-*β*-CD. Results are presented as the means of six different experiments ± standard deviation.

On the basis of these interesting results, a preliminary ex vivo study able to evaluate the effect of HP-*β*-CD on nasal permeation of IDE was performed. Excised bovine nasal mucosa was mounted on Franz-type diffusion cells, and the receptor phase was sampled within 4 h to determine permeated IDE. A suspension of the free drug at 30 µM dose was assayed compared to a solution of the 1:2 IDE/HP-*β*-CD inclusion complex at the same dose, and with solutions of IDE (30 µM) in the presence of excess amounts of HP-*β*-CD. As observed in Figure 20, a progressive enhancement of the permeated IDE was observed by increasing the macrocycle concentration. As a first approximation, one can hypothesize that the enhancement of IDE permeation could have been due to the enhanced water solubility of the drug produced by complexation. The latter, in turn, increased the contact of IDE with the nasal mucosa. However, this cannot be the only explanation, since excess amounts of HP-*β*-CD added to IDE produced a further increase in permeation, as can be seen in Figure 20. Thus, it is evident that the ability of HP-*β*-CD to interact with the biomembrane components [34], altering the permeability of nasal mucosa, played also a role in IDE permeation. On the other hand, a previous work [84] demonstrated that high concentrations of HP-*β*-CD increased the permeation of papaverine through the skin without influencing the water solubility of the included drug.

As a matter of fact, an inspection of Figure 20 suggests that the direct interaction of HP-*β*-CD with the nasal mucosa seemed to play a minor role in IDE permeation other than the solubilizing effect. In fact, at the end of the experiment, the IDE permeation was shown to pass from about 1% in the case of free drug to about 5% when complexed in a 1:2 molar ratio. Once inclusion occurred, on the other side, the further enhancement of permeated IDE observed after the addition of an excess of CD appeared less relevant.

**Figure 21:**
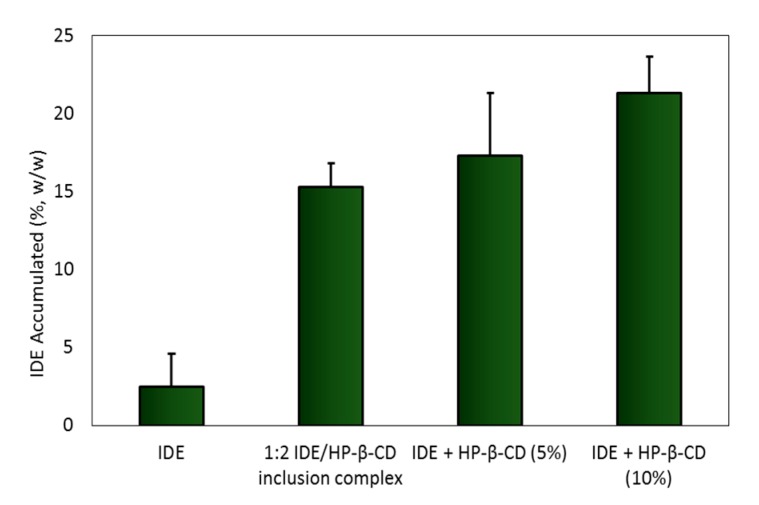
Amounts of IDE accumulated into the excised bovine nasal mucosa at the end of the permeation experiment (4 h). Results are presented as the means of six different experiments ± standard deviation.

As a consequence of the increased permeability of IDE, we also observed a significant percentage of IDE accumulated in the mucosa (Figure 21), confirming the important role played by the macrocycle in the IDE uptake by biological barriers [85].

## 4. Conclusions

An extensive spectroscopic investigation of the IDE/HP-*β*-CD inclusion complex was here performed to provide valuable information in order to clarify the complexation mechanism and the geometry. Moreover, solubility studies and biological assays were performed.

In the solid phase, SEM, as well as FTIR-ATR with mid-infrared light, FT-NIR, and Raman spectroscopy results, gave evidence of the formation of the inclusion complex and allowed for the identification of the functional groups involved in the host–guest interactions.

Particular attention was paid to the analysis of the O–H stretching mode of the complex, which evidenced, vs. T, the existence of an isosbestic point. Through deconvolution and curve-fitting of this band, the average enthalpy value Δ*H*_HB_ of the H-bond scheme upon inclusion was obtained.

In the liquid state, NMR analysis evidenced the formation of the IDE/HP-*β*-CD inclusion complex, showing a downfield displacement of the IDE protons when complexed with the macrocycle, evidencing their proximity to an electronegative atom, like oxygen. The 2D ROESY analysis and computational studies demonstrated the inclusion of the quinone ring of IDE inside the CD ring. The IDE alkyl chain points out of the CD and is aligned with the tethered 2-hydroxypropyl groups. The binding energy was about −5 kcal/mol and was in good agreement with the experimental one (*K*_c_ = 6031 M^−1^).

The water solubility and dissolution rate of IDE strongly increased as a result of complexation, demonstrating the good potentiality of HP-*β*-CD as a delivery system for IDE. Biological in vitro studies showed a high protective effect for the IDE/HP-*β*-CD inclusion complex, with a clear dose-dependent profile, against the hydrogen peroxide-induced cell damage. Ex vivo studies on excised bovine nasal mucosa evidenced high permeation of IDE when the macrocycle was present. This result was due both to the solubilizing effect produced by complexation and, to a lesser extent, the penetration enhancer activity exerted by HP-*β*-CD, thus suggesting a potential role of the IDE/HP-*β*-CD inclusion complex in improving the therapeutic performance of IDE.

## Figures and Tables

**Table 1:** ^1^H-NMR chemical shift assignments for free idebenone (IDE) and the IDE/2-hydroxypropyl-*β*-cyclodextrin (HP-*β*-CD) complex.

Proton	IDE	IDE/HP-*β* -CD	Δ*δ*
1	3.48	3.52	0.04
2	1.46	1.49	0.03
3	2.39	2.43	0.04
4	1.92	1.98	0.06
5,6	3.89	3.93	0.04

Δ*δ* = *δ*_complex_ − *δ*_free_.

## Supplementary Materials

The following are available online at https://www.mdpi.com/2218-273X/9/10/531/s1: Figure S1: Plot of peak area versus different IDE concentrations; Figure S2: ^1^H NMR spectrum of HP-*β*-CD; Figure S3: ^1^H NMR spectrum of IDE; Figure S4: ^1^H NMR spectrum of IDE/HP-*β*-CD inclusion complex; Figure S5: 2D ROESY plot of IDE/HP-*β*-CD complex in D_2_O/MeOD.

## References

[1] Jaber S., Polster B.M. (2015). Idebenone and neuroprotection: Antioxidant, pro-oxidant, or electron carrier?. J. Bioenerg. Biomembr..

[2] Attia H.A., AL-Rasheed N.M., Faddah L.M., AL-Rasheed N.M., Ahmed A.A. (2009). Ameliorating effect of idebenone and/or melatonin against oxidative stress and energy depletion in brain of hypoxic rats. Res. J. Med. Med. Sci..

[3] Zs-Nagy I. (1990). Chemistry, toxicology, pharmacology and pharmacokinetics of idebenone: A review. Arch. Gerontol. Geriatr..

[4] Barroso Rodríguez N.S., Santos Caballero N., Ramírez Jasso J.A., Aguilar Nava J. (2001). Idebenone in patients with cognitive disorders following stroke. Invest. Méd. Int..

[5] Yan A., Liu Z., Song L., Wang X., Zhang Y., Wu N., Lin J., Liu Y., Liu Z. (2019). Idebenone Alleviates Neuroinflammation and Modulates Microglial Polarization in LPS-Stimulated BV2 Cells and MPTP-Induced Parkinson’s Disease Mice. Front. Cell. Neurosci..

[6] Schaffler K., Hadler D., Stark M. (1998). Dose-effect relationship of idebenone in an experimental cerebral deficit model. Pilot study in healthy young volunteers with piracetam as reference drug. Arzneimittelforschung.

[7] Montenegro L., Turnaturi R., Parenti C., Pasquinucci L. (2018). Idebenone: Novel Strategies to Improve Its Systemic and Local Efficacy. Nanomaterials.

[8] Thal L.J., Grundman M., Berg J., Ernstrom K., Margolin R., Pfeiffer E., Weiner M.F., Zamrini E., Thomas R.G. (2003). Idebenone treatment fails to slow cognitive decline in Alzheimer’s disease. Neurology.

[9] Gillis J.C., Benfield P., McTavish D. (1994). Idebenone. A review of its pharmacodynamic and pharmacokinetic properties, and therapeutic use in age-related cognitive disorders. Drugs Aging.

[10] Lyseng-Williamson K.A. (2016). Idebenone: A Review in Leber’s Hereditary Optic Neuropathy. Drugs.

[11] Carelli V., Carbonelli M., de Coo I., Kawasaki A., Klopstock T., Lagrèze W., La Morgia C., Newman N., Orssaud C., Pott J.W. (2017). International Consensus Statement on the Clinical and Therapeutic Management of Leber Hereditary Optic Neuropathy. J. Neuro-Ophthalmol..

[12] Orssaud C., Bidot S., Lamirel C., Brémond Gignac D., Touitou V., Vignal C. (2019). Raxone in the Leber optical neuropathy: Parisian experience. J. Fr. Ophtalmol..

[13] Di Prospero N.A., Baker A., Jeffries N., Fischbeck K.H. (2007). Neurological effects of high-dose idebenone in patients with Friedreich’s ataxia: A randomised, placebo-controlled trial. Lancet Neurol..

[14] Brandsema J.F., Stephens D., Hartley J., Yoon G. (2010). Intermediate-dose idebenone and quality of life in Friedreich ataxia. Pediatr. Neurol..

[15] Kearney M., Orrell R.W., Fahey M., Brassington R., Pandolfo M. (2016). Pharmacological treatments for Friedreich ataxia. Cochrane Database Syst. Rev..

[16] Cook A., Boesch S., Heck S., Brunt E., Klockgether T., Schöls L., Schulz A., Giunti P. (2019). Patient-reported outcomes in Friedreich’s ataxia after withdrawal from idebenone. Acta Neurol. Scand..

[17] Lynch D.R., Perlman S.L., Meier T. (2010). A phase 3, double-blind, placebo-controlled trial of idebenone in friedreich ataxia. Arch. Neurol..

[18] Buyse G.M., Voit T., Schara U., Straathof C.S.M., D’Angelo M.G., Bernert G., Cuisset J.M., Finkel R.S., Goemans N., McDonald C.M. (2015). Efficacy of idebenone on respiratory function in patients with Duchenne muscular dystrophy not using glucocorticoids (DELOS): A double-blind randomised placebo-controlled phase 3 trial. Lancet.

[19] McDonald C.M., Meier T., Voit T., Schara U., Straathof C.S., D’Angelo M.G., Bernert G., Cuisset J.M., Finkel R.S., Goemans N. (2016). Idebenone reduces respiratory complications in patients with Duchenne muscular dystrophy. Neuromuscul. Disord..

[20] Bodmer M., Vankan P., Dreier M., Kutz K.W., Drewe J. (2009). Pharmacokinetics and metabolism of idebenone in healthy male subjects. Eur. J. Clin. Pharmacol..

[21] Mistry A., Stolnik S., Illum L. (2009). Nanoparticles for direct nose-to-brain delivery of drugs. Int. J. Pharm..

[22] Bourganis V., Kammona O., Alexopoulos A., Kiparissides C. (2018). Recent advances in carrier mediated nose-to-brain delivery of pharmaceutics. Eur. J. Pharm. Biopharm..

[23] Lochhead J.J., Thorne R.G. (2012). Intranasal delivery of biologics to the central nervous system. Adv. Drug Deliv. Rev..

[24] Na L., Mao S., Wang J., Sun W. (2010). Comparison of different absorption enhancers on the intranasal absorption of isosorbide dinitrate in rats. Int. J. Pharm..

[25] Chavanpatil M., Vavia P.R. (2002). Enhancement of Nasal Absorption of Acyclovir via Cyclodextrins. J. Incl. Phenom. Macrocycl. Chem..

[26] Brewster M.E., Loftsson T. (2007). Cyclodextrins as pharmaceutical solubilizers. Adv. Drug Deliv. Rev..

[27] Stancanelli R., Crupi V., De Luca L., Ficarra P., Ficarra R., Gitto R., Guardo M., Iraci N., Majolino D., Tommasini S. (2008). Improvement of water solubility of non-competitive AMPA receptor antagonists by complexation with beta-cyclodextrin. Bioorg. Med. Chem..

[28] Venuti V., Stancanelli R., Acri G., Crupi V., Paladini G., Testagrossa B., Tommasini S., Ventura C.A., Majolino D. (2017). “Host-guest” interactions in Captisol^®^/Coumestrol inclusion complex: UV-vis, FTIR-ATR and Raman studies. J. Mol. Struct..

[29] Lopez R.F.L., Collett J.H., Bentley M.V.L.B. (2000). Influence of cyclodextrin complexation on the in vitro permeation and skin metabolism of dexamethasone. Int. J. Pharm..

[30] Cannavà C., Crupi V., Guardo M., Majolino D., Stancanelli R., Tommasini S., Ventura C.A., Venuti V. (2013). Phase solubility and FTIR-ATR studies of idebenone/sulfobutylether-β-cyclodextrin inclusion complex. J. Incl. Phenom. Macrocycl. Chem..

[31] Puglisi G., Ventura C.A., Fresta M., Vandelli M.A., Cavallaro G., Zappalà M. (1996). Preparation and physico-chemical study of inclusion complexes between idebenone and modified β-cyclodextrins. J. Incl. Phenom. Mol. Recognit. Chem..

[32] Lauro F., Ilari S., Giancotti L.A., Ventura C.A., Morabito C., Gliozzi M., Malafoglia V., Palma E., Paolino D., Mollace V. (2016). Pharmacological effect of a new idebenone formulation in a model of carrageenan-induced inflammatory pain. Pharm. Res..

[33] López-Nicolás J.M., Rodríguez-Bonilla P., García-Carmona F. (2014). Cyclodextrins and antioxidants. Crit. Rev. Food Sci. Nutr..

[34] Di Cagno M.P. (2017). The Potential of Cyclodextrins as Novel Active Pharmaceutical Ingredients: A Short Overview. Molecules.

[35] Higuchi T., Connors K.A. (1965). Phase solubility techniques. Adv. Anal. Chem. Instrum..

[36] Maréchal Y. (2003). Observing the water molecule in macromolecules and aqueous media using infrared spectrometry. J. Mol. Struct..

[37] Gans P., Sabatini A., Vacca A. (2008). Protonic Software Leeds, UK. http://www.hyperquad.co.uk/HQ2008.htm.

[38] Gans P., Sabatini A., Vacca A. (1996). Investigation of equilibria in solution. Determination of equilibrium constants with the HYPERQUAD suite of programs. Talanta.

[39] Gans P., Sabatini A., Vacca A. (1999). Determination of equilibrium constants from spectrophometric data obtained from solutions of known pH: The program pHab. Ann. Chim..

[40] Shrivastava A., Gupta V.B. (2011). Methods for the determination of limit of detection and limit of quantitation of the analytical methods. Chron. Young Sci..

[41] Zhang C.L., Liu J.C., Yang W.B., Chen D.L., Jiao Z.G. (2017). Experimental and molecular docking investigations on the inclusion mechanism of the complex of phloridzin and hydroxypropyl-beta-cyclodextrin. Food Chem..

[42] Krieger E., Vriend G. (2014). YASARA View-molecular graphics for all devices-from smartphones to workstations. Bioinformatics.

[43] Land H., Humble M.S. (2018). YASARA: A Tool to Obtain Structural Guidance in Biocatalytic Investigations. Methods Mol. Biol..

[44] Duan Y., Wu C., Chowdhury S., Lee M.C., Xiong G., Zhang W., Yang R., Cieplak P., Luo R., Lee T. (2003). A point-charge force field for molecular mechanics simulations of proteins based on condensed-phase quantum mechanical calculations. J. Comput. Chem..

[45] Jakalian A., Jack D.B., Bayly C.I. (2002). Fast, efficient generation of high-quality atomic charges. AM1-BCC model: II. Parameterization and validation. J. Comput. Chem..

[46] Wongpituk P., Nutho B., Panman W., Kungwan N., Wolschann P., Rungrotmongkol T., Nunthaboot N. (2017). Structural dynamics and binding free energy of neral-cyclodextrins inclusion complexes: Molecular dynamics simulation. Mol. Simul..

[47] Genheden S., Ryde U. (2015). The MM/PBSA and MM/GBSA methods to estimate ligand-binding affinities. Expert Opin. Drug Discov..

[48] Cosco D., Failla P., Costa N., Pullano S., Fiorillo A., Mollace V., Fresta M., Paolino D. (2016). Rutin-loaded chitosan microspheres: Characterization and evaluation of the anti-inflammatory activity. Carbohydr. Polym..

[49] Paolino D., Cosco D., Celano M., Moretti S., Puxeddu E., Russo D., Fresta M. (2013). Gemcitabine-loaded biocompatible nano capsules for the effective treatment of human cancer. Nanomedicine (Lond).

[50] Rajamohan R., Kothainayaki S., Swaminatan M. (2008). Spectrofluorimetricstudy on inclusion complexation of 2-amino-6-fluorobenzothiazole with β-cyclodextrin. Collect. Czechoslov. Chem. Commun..

[51] Crupi V., Ficarra R., Guardo M., Majolino D., Stancanelli R., Venuti V. (2007). UV–vis and FTIR–ATR spectroscopic techniques to study the inclusion complexes of genistein with β-cyclodextrins. J. Pharm. Biomed. Anal..

[52] Brubach J.B., Mermet A., Filabozzi A., Gerschel A., Lairez D., Krafft M.P., Roy P. (2001). Dependence of water dynamics upon confinement size. J. Phys. Chem. B.

[53] Crupi V., Majolino D., Mele A., Melone L., Punta C., Rossi B., Toraldo F., Trotta F., Venuti V. (2014). Direct evidence of gel-sol transition in cyclodextrin-based hydrogels as revealed by FTIR-ATR spectroscopy. Soft Matter.

[54] Gavira J.M., Hernanz A., Bratu I. (2003). Dehydration of β-cyclodextrin: An IR ν(OH) band profile analysis. Vib. Spectrosc..

[55] Venuti V., Rossi B., Crupi V., D’Amico F., Gessini A., Majolino D., Masciovecchio C., Stancanelli R., Ventura C.A. (2016). Solute-solvent interactions in aqueous solutions of sulfobutyl ether-β-cyclodextrin as probed by UV-Raman and FTIR-ATR analysis. J. Phys. Chem. B.

[56] Crupi V., Longo F., Majolino D., Venuti V. (2007). Raman spectroscopy: Probing dynamics of water molecules confined in nanoporous silica glasses. Eur. Phys. J. Spec. Top..

[57] Bratu I., Veiga F., Fernandes C., Hernanz A., Gavira J.M. (2004). Infrared spectroscopic study of triacetyl-β-cyclodextrin and its inclusion complex with nicapiridine. Spectroscopy.

[58] Castiglione F., Crupi V., Majolino D., Mele A., Rossi B., Trotta F., Venuti V. (2012). Effect of cross-linking properties on the vibrational dynamics of cyclodextrins-based polymers: An experimental–numerical study. J. Phys. Chem. B.

[59] Stancanelli R., Venuti V., Arigò A., Calabrò M.L., Cannavà C., Crupi V., Majolino D., Tommasini S., Ventura C.A. (2015). Isoflavone aglycons-sulfobutyl ether-β-cyclodextrin inclusion complexes: In solution and solid state studies. J. Incl. Phenom. Macrocycl. Chem..

[60] Crupi V., Maisano G., Majolino D., Migliardo P., Venuti V. (2000). Anharmonic effects and vibrational dynamics in h-bonded liquids by attenuated total reflectance FT-IR spectroscopy. J. Phys. Chem. A.

[61] Gallina M.E., Sassi P., Paolantoni M., Morresi A., Cataliotti R.S. (2006). Vibrational analysis of molecular interactions in aqueous glucose solutions. Temperature and concentration effects. J. Phys. Chem. B.

[62] Celia C., Scala A., Stancanelli R., Surdo E., Paolino D., Grattoni A., Micale N., Crupi V., Majolino D., Fresta M. (2007). Physicochemical properties of inclusion complexes of highly soluble β-cyclodextrins with highly hydrophobic testosterone propionate. Int. J. Pharm..

[63] Crupi V., Majolino D., Mele A., Rossi B., Trotta F., Venuti V. (2013). Modelling the interplay between covalent and physical interactions in cyclodextrin-based hydrogel: Effect of water confinement. Soft Matter.

[64] Jug M., Bećirević-Laćan M., Kwokal A., Cetina-Čižmek B. (2005). Influence of cyclodextrin complexation on piroxicam gel formulations. Acta Pharm..

[65] Mohan P.R.K., Sreelakshmi G., Muraleedharan C.V., Joseph R. (2012). Water soluble complexes of curcumin with cyclodextrins: Characterization by FT-Raman spectroscopy. Vib. Spectrosc..

[66] Sancho M.I., Andujar S., Porasso R.D., Enriz R.D. (2016). Theoretical and experimental study of inclusion complexes of β-cyclodextrins with chalcone and 2′,4′-dihydroxychalcone. J. Phys. Chem. B.

[67] Hibberta D.B., Thordarson P. (2016). The death of the job plot, transparency, open science and online tools, uncertainty estimation methods and other developments in supramolecular chemistry data analysis. Chem. Commun..

[68] Ulatowski F., Dąbrowa K., Bałakier T., Jurczak J. (2016). Recognizing the limited applicability of job plots in studying host-guest interactions in supramolecular chemistry. J. Org. Chem..

[69] Floresta G., Rescifina A. (2019). Metyrapone-β-cyclodextrin supramolecular interactions inferred by complementary spectroscopic/spectrometric and computational studies. J. Mol. Struct..

[70] Damiani E., Yuecel R., Wallace H.M. (2019). Repurposing of Idebenone as a potential anticancer agent. Biochem. J..

[71] Tai K.K., Pham L., Truong D.D. (2011). Idebenone Induces Apoptotic Cell Death in the Human Dopaminergic Neuroblastoma SHSY-5Y Cells. Neurotox. Res..

[72] Loftsson T., Jarho P., Masson M., Jarvinen T. (2005). Cyclodextrins in drug delivery. Expert Opin. Drug Deliv..

[73] Costa P., Medronho B., Gonḉalves S., Romano A. (2015). Cyclodextrins enhance the antioxidant activity of essential oils fromthree Lamiaceae species. Ind. Crop. Prod..

[74] Kim H., Yiluo H., Park S., Lee J.Y., Cho E., Jung S. (2016). Characterization and Enhanced Antioxidant Activity of the Cysteinyl β-Cyclodextrin-Baicalein Inclusion Complex. Molecules.

[75] Alvarez-Parrilla E., De La Rosa L.A., Torres-Rivas F., Rodrigo-Garcia J., Gonzalez-Aguilar G. (2005). Complexation of Apple Antioxidants: Chlorogenic Acid, Quercetin and Rutin by β-Cyclodextrin (β-CD). J. Incl. Phenom. Macrocycl. Chem..

[76] Roy P., Dinda A.K., Chaudhury S., Dasgupta S. (2018). β-cyclodextrin encapsulated polyphenols as effective antioxidants. Biopolymers.

[77] Li S., Yuan L., Chen Y., Zhou W., Wang X. (2017). Studies on the Inclusion Complexes of Daidzein with β-Cyclodextrin and Derivatives. Molecules.

[78] Chouhan P., Saini T.R. (2014). Hydroxypropyl-β-cyclodextrin: A Novel Transungual Permeation Enhancer for Development of Topical Drug Delivery System for Onychomycosis. J. Drug Deliv..

[79] Jansook P., Ogawa N., Loftsson T. (2018). Cyclodextrins: Structure, physicochemical properties and pharmaceutical applications. Int. J. Pharm..

[80] Coisne C., Tilloy S., Monflier E., Wils D., Fenart L., Gosselet F. (2016). Cyclodextrins as Emerging Therapeutic Tools in the Treatment of Cholesterol-Associated Vascular and Neurodegenerative Diseases. Molecules.

[81] Zimmer S., Grebe A., Bakke S.S., Bode N., Halvorsen B., Ulas T., Skjelland M., De Nardo D., Labzin L.I., Kerksiek A. (2016). Cyclodextrin promotes atherosclerosis regression via macrophage reprogramming. Sci. Transl. Med..

[82] Kim T.K., Kang W., Chun I.K., Oh S.Y., Lee Y.H., Gwak H.S. (2009). Pharmacokinetic evaluation and modeling of formulated levodopa intranasal delivery systems. Eur. J. Pharm. Sci..

[83] Rathi A.A., Dhamecha D.L., Patel K.A., Saifee M., Dehghan M.H.G. (2011). Effect of permeation enhancers on permeation kinetics of idebenone through the bovine buccal mucosa. Indian J. Pharm. Educ. Res..

[84] Ventura C.A., Fresta M., Paolino D., Pedotti S., Corsaro A., Puglisi G. (2001). Biomembrane model interaction and percutaneous absorption of papaverine through rat skin: Effects of cyclodextrins as penetration enhancers. J. Drug Target..

[85] Loftsson T., Brewster M.E. (2011). Pharmaceutical applications of cyclodextrins: Effects on drug permeation through biological membranes. J. Pharm. Pharm..

