# Supplementary material for: Physicochemical Characterization and Antioxidant Activity Evaluation of Idebenone/Hydroxypropyl-β-Cyclodextrin Inclusion Complex
Source: Biomolecules. 2019 Sep 25;9(10):531. doi: 10.3390/biom9100531 (PMC6843366; doi:10.3390/biom9100531)
Supplement: Supplementary file 1 [file biomolecules-09-00531-s001.pdf]

biomolecules

Supplementary Materials

Physicochemical Characterization and Antioxidant Activity Evaluation of Idebenone/Hydroxypropyl-*β*-Cyclodextrin Inclusion Complex †

Valentina Venuti [1], Vincenza Crupi [2], Barbara Fazio [3], Domenico Majolino [1,*], Giuseppe Acri [4], Barbara Testagrossa [4], Rosanna Stancanelli [2], Federica De Gaetano [2], Agnese Gagliardi [5], Donatella Paolino [5], Giuseppe Floresta [6], Venerando Pistarà [6], Antonio Rescifina [6] and Cinzia A. Ventura [2,*]

1 Dipartimento di Scienze Matematiche e Informatiche, Scienze Fisiche e Scienze della Terra, Università degli Studi di Messina, V.le F. Stagno D'Alcontres, 31-98166 Messina, Italy; vvenuti@unime.it
2 Dipartimento di Scienze Chimiche, Biologiche, Farmaceutiche e Ambientali, Università degli Studi di Messina, V.le F. Stagno D'Alcontrés, 31-98166 Messina, Italy; vcrupi@unime.it (V.C.), rstancanelli@unime.it (R.S.), fedegaetano@unime.it (F.D.G.)
3 CNR-IPCF Istituto per i Processi Chimico Fisici, V.le F. Stagno d'Alcontres, 37-98158 Faro Superiore, Messina, Italy; fazio@me.cnr.it
4 Dipartimento di Scienze Biomediche, Odontoiatriche, e delle Immagini Morfologiche e Funzionali, Università degli Studi di Messina, c/o A.O.U. Policlinico "G. Martino" Via Consolare Valeria, 1 - 98125 Messina, Italy; gacri@unime.it (G.A.), btestagrossa@unime.it (B.T.)
5 Dipartimento di Medicina Clinica e Sperimentale, Università degli Studi di Catanzaro "Magna Græcia", Campus Universitario "S. Venuta", Viale S. Venuta-88100 Germaneto, Catanzaro, Italy; gagliardi@unicz.it (A.G.), paolino@unicz.it (D.P.)
6 Dipartimento di Scienze del Farmaco, Università degli Studi di Catania, V.le A. Doria, 6-95125 Catania, Italy; giuseppe.floresta@unict.it (G.F.), vpistara@unict.it (V.P.), arescifina@unict.it (A.R.)
* Correspondence: dmajolino@unime.it (D.M.); caventura@unime.it (C.A.V.)

† In memory of Professor Carmela Spatafora, friend, colleague and distinguished scientist, on the third anniversary of her premature death.

Analytical Method Validation

The calibration curve of IDE was linear in the range 0.2-50 μg/mL (R2 = 0.9997) (Figure S1). No interference was produced by different amounts of HP-β-CD on retention time of IDE (RSD, 0.52%) and peak area (RSD, 0.65%), demonstrating method to be specific for the drug. LOD and LOQ were 0.06 μg/mL (corresponding to 3:1 signal-to-noise ratio) and 0.2 μg/mL (corresponding to 10:1 signal-to-noise ratio), respectively. The method showed high intraday (RSD, 0.76%) and interday (RSE, 0.43%) precision. No degradation of IDE was observed during analysis.

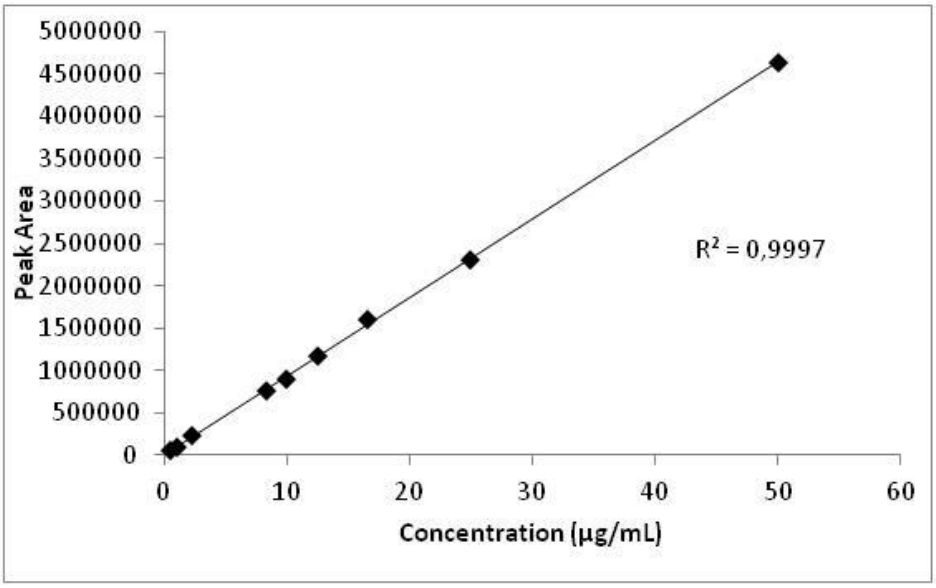

Figure 1. Plot of peak area vs. IDE concentration.

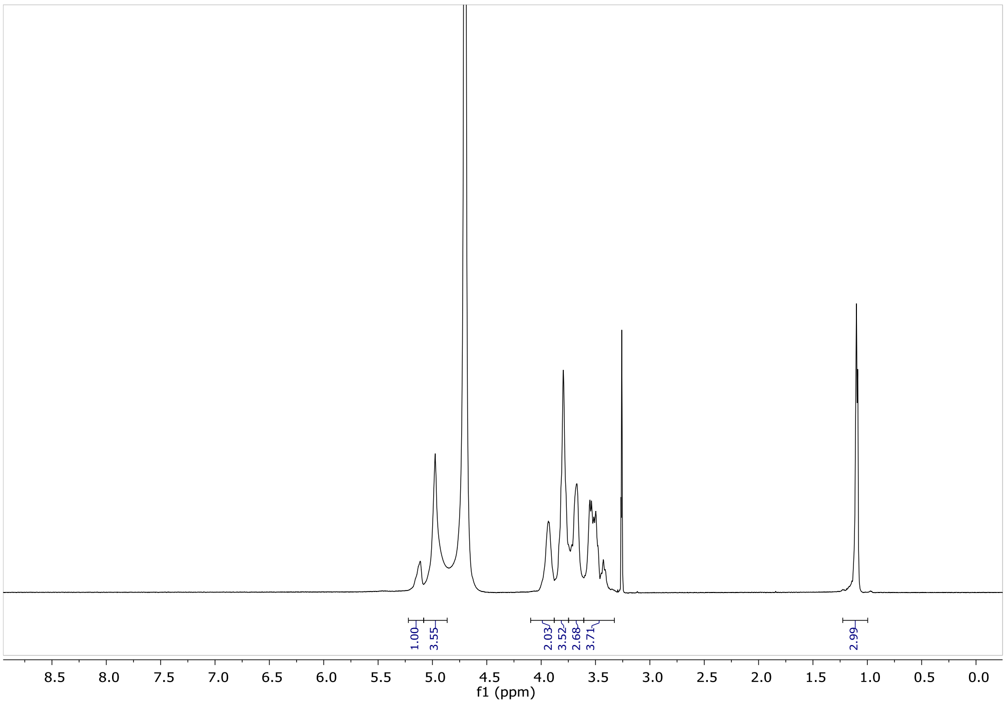

Figure 2. ^{1}H NMR spectrum of HP-*β*-CD.

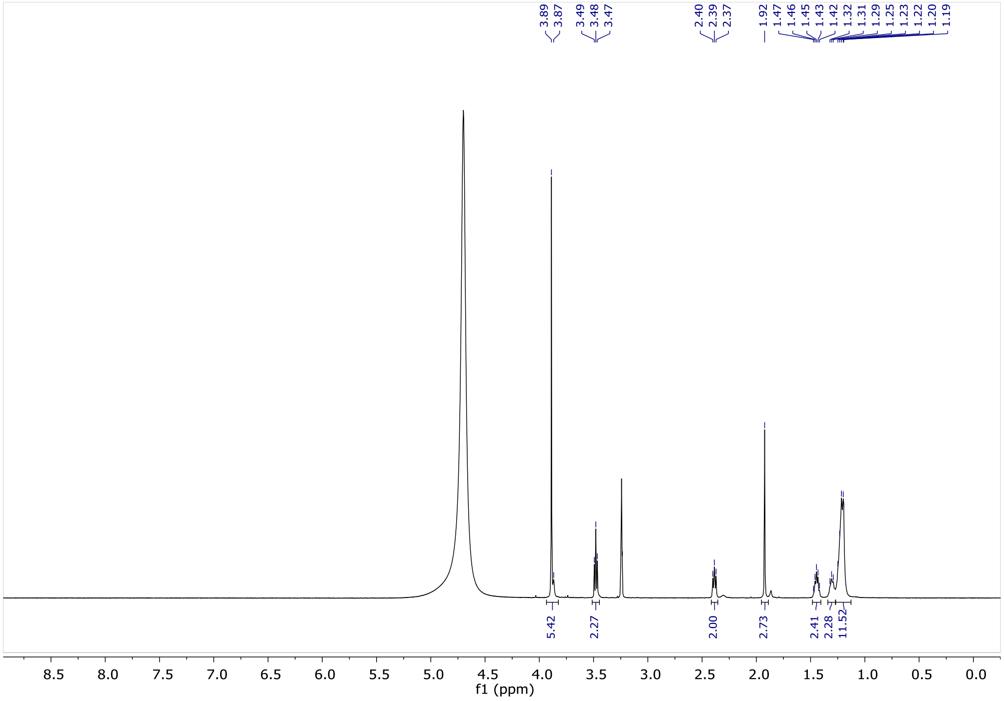

Figure 3. ^{1}H NMR spectrum of IDE.

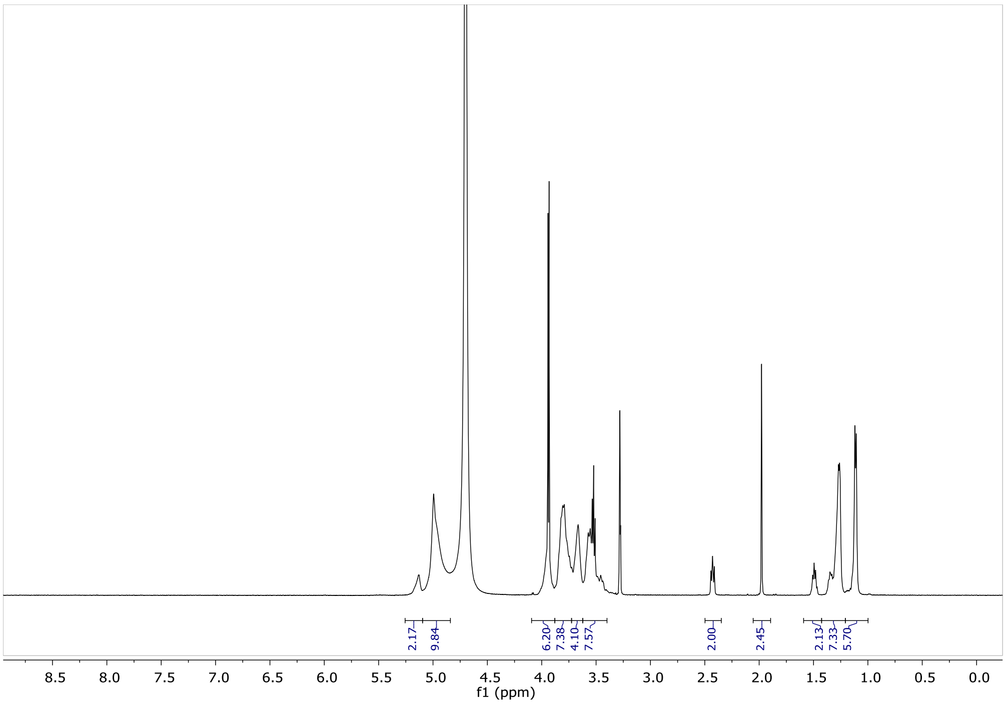

Figure 4. ^{1}H NMR spectrum of IDE/HP-*β*-CD inclusion complex.

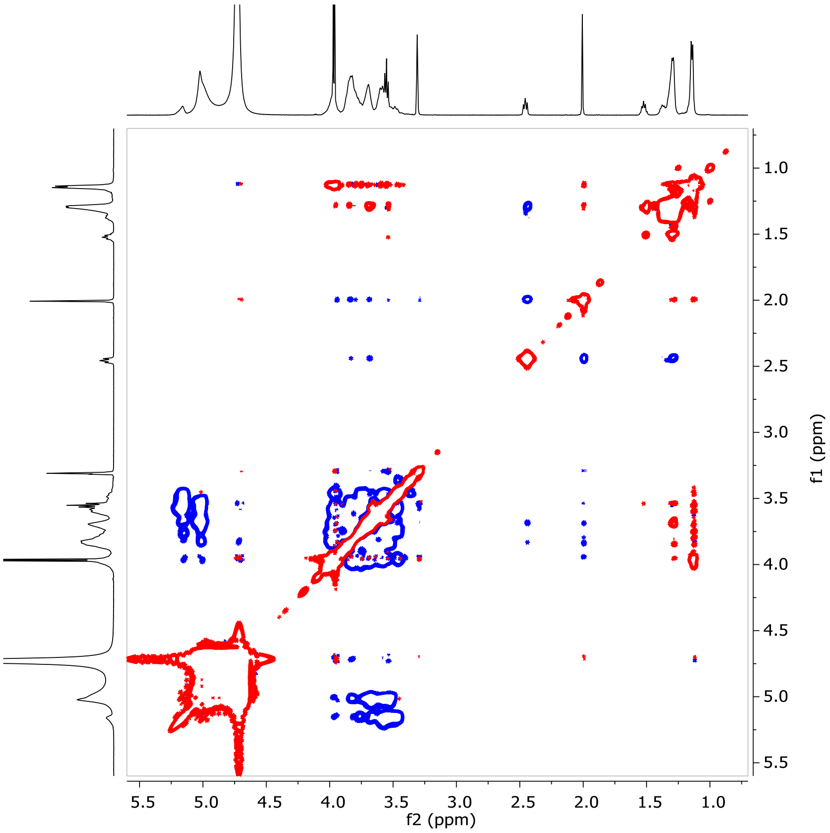

Figure 5. 2D ROESY plot of IDE/HP-*β*-CD complex in D_2O/MeOD.

© 2019 by the authors. Licensee MDPI, Basel, Switzerland. This article is an open access article distributed under the terms and conditions of the Creative Commons Attribution (CC BY) license (http://creativecommons.org/licenses/by/4.0/).
